# Supplementary material for: C1-linker region of PARG1 RhoGAP promotes the catalytic recognition fold of RhoA substrate
Source: PLoS One. 2025 Jul 9;20(7):e0326924. doi: 10.1371/journal.pone.0326924 (PMC12240320; doi:10.1371/journal.pone.0326924)

Binding free energy calculation

a) PARG1 GAP domain

WT PARG1#

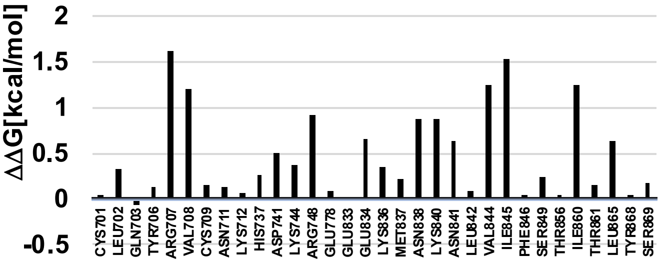

WT PARG1##

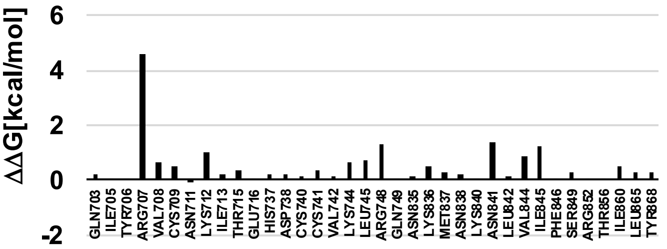

PARG1 I845V

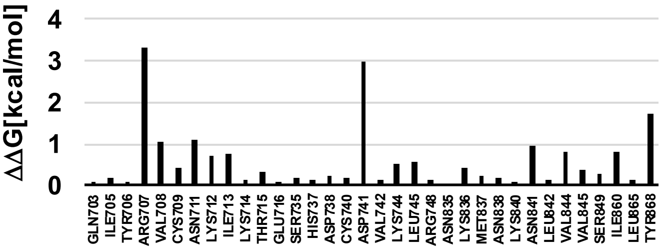

PARG1 T622M

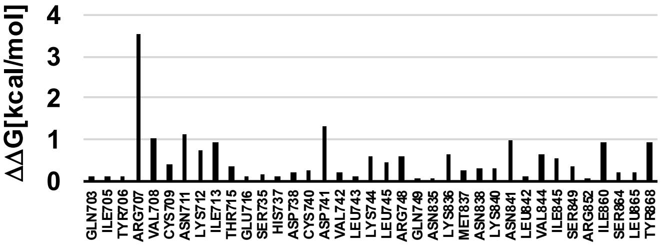

b) RhoGTPase

RhoA (WT PARG1#)

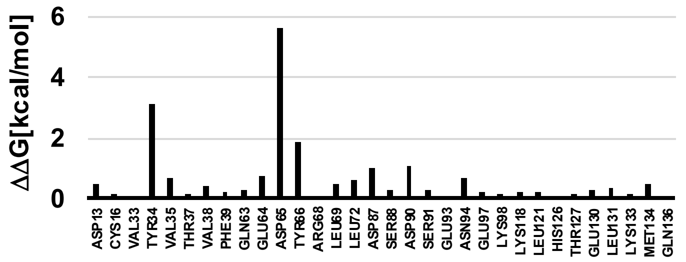

RhoA (WT PARG1##)

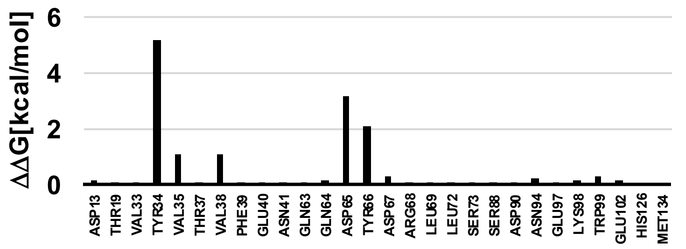

RhoA (PARG1 I845V)

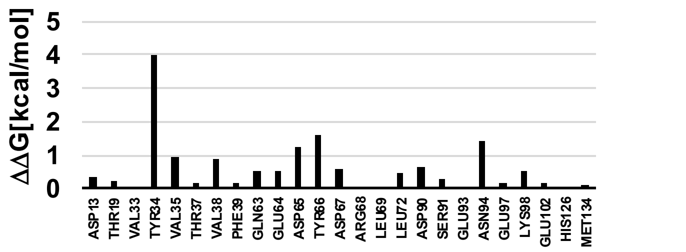

RhoA (PARG1 T622M)

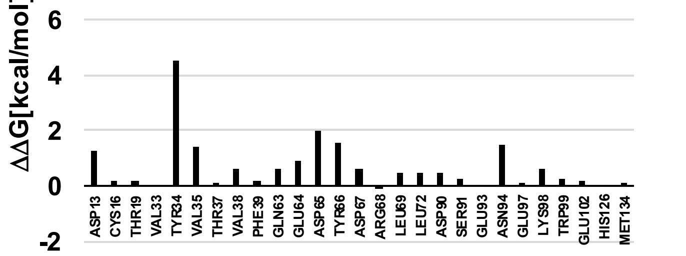

Degree of Buriedness

c) PARG1 GAP domain

WT PARG1#

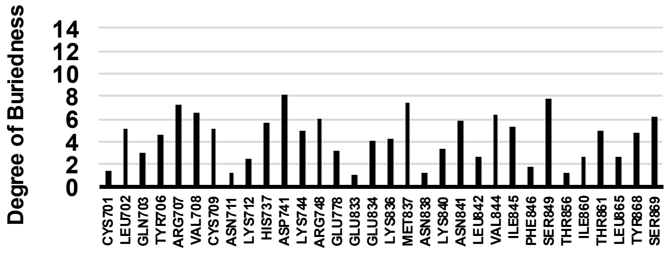

WT PARG1##

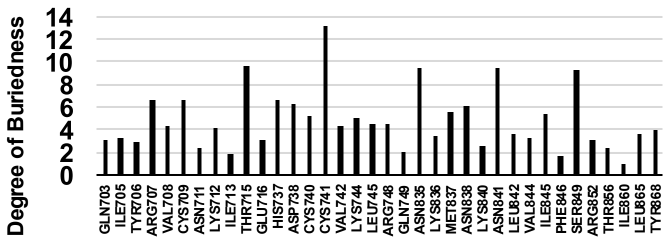

PARG1 I845V

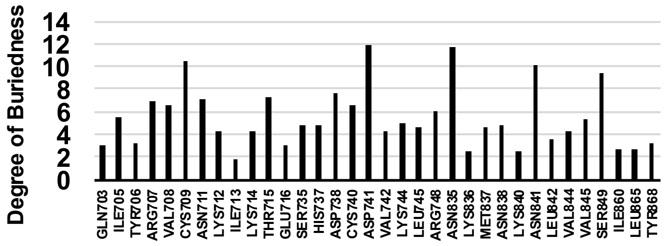

PARG1 T622M

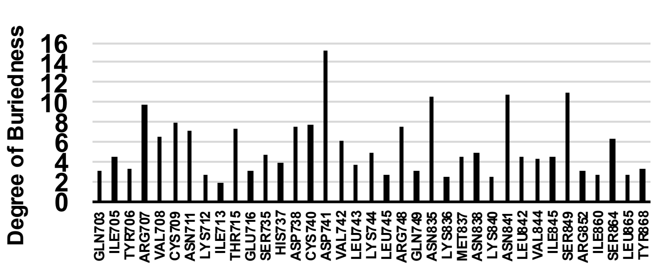

d) RhoGTPase

RhoA (WT PARG1#)

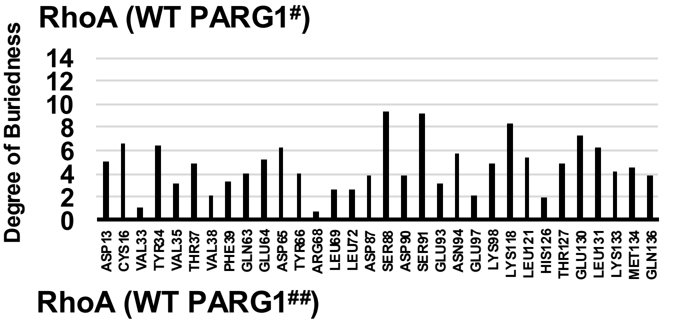

RhoA (WT PARG1##)

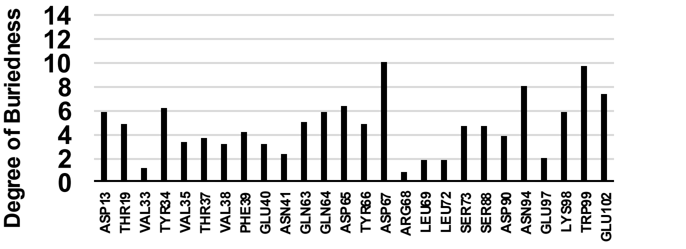

RhoA (PARG1 I845V)

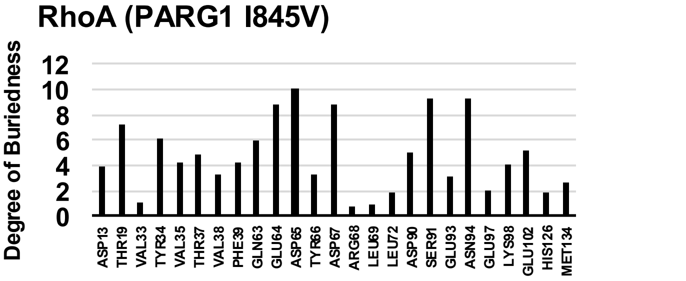

RhoA (PARG1 T622M)

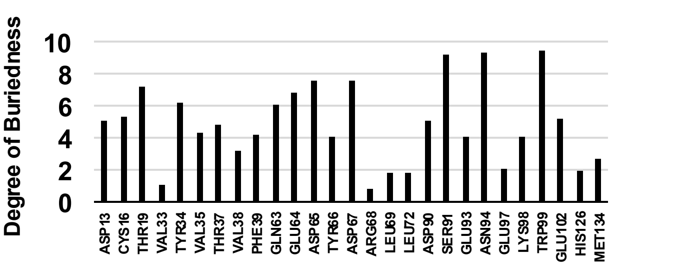

Supplement: S1 Fig — Interacting residues in RhoGAP domains (a, c) of human WT PARG1# (residue number: 658−898), WT PARG1## containing the C1 domain (residue number: 611−886), I845V mutant, and T622M mutant interacting with RhoA (b, d) (PDB code: 5irc for PARG1#; 5c2k for PARG1##, I845V, and T622M mutants) were examined by DrugScorePPI software program. Each of the interacting residues individually mutated to alanine was scored by the change in binding free energy (ΔΔG) (a, b) and the degree of buriedness of each residue (c, d) in the interface was calculated. The residue number of target proteins is shown according to the modeled domain structure (Q52LW3−1). (PDF) [file pone.0326924.s001.pdf]
