# Supplementary figures and images for: C1-linker region of PARG1 RhoGAP promotes the catalytic recognition fold of RhoA substrate
Source: PLoS One. 2025 Jul 9;20(7):e0326924. doi: 10.1371/journal.pone.0326924 (PMC12240320; doi:10.1371/journal.pone.0326924)

### PARG1#(241aa) 1ow3

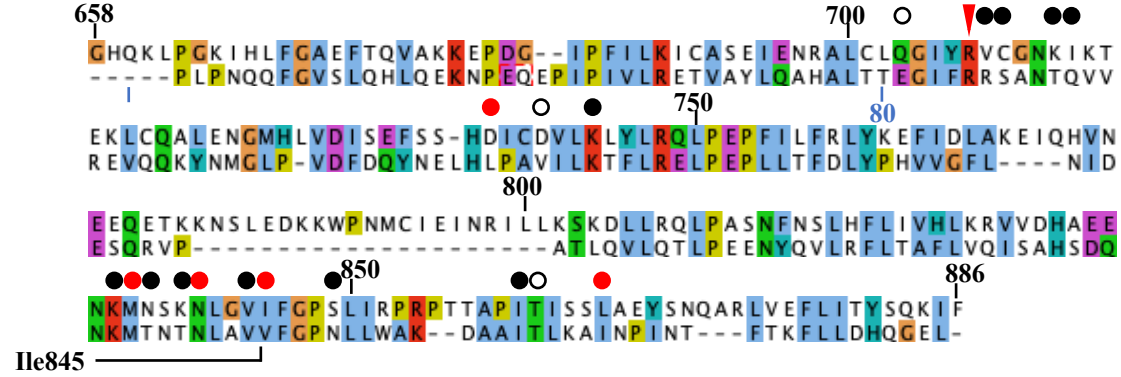

### PARG1# 3msx

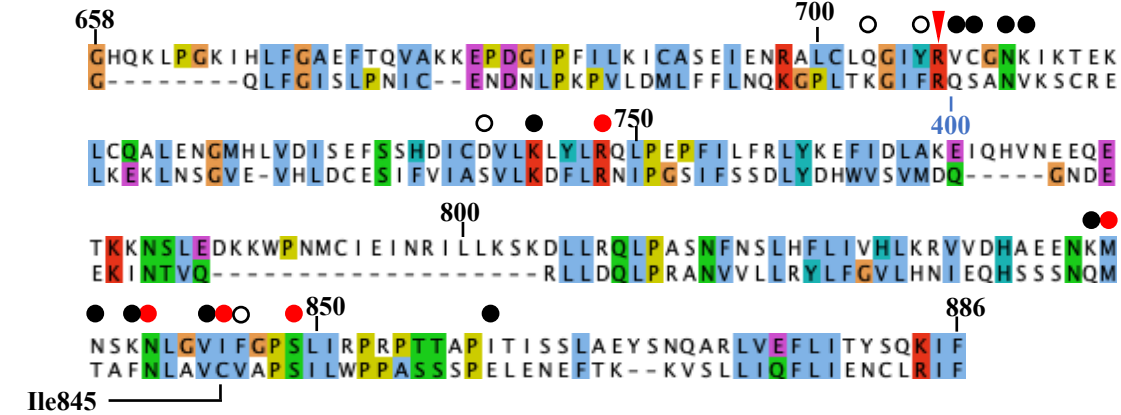

### PARG1# 5irc

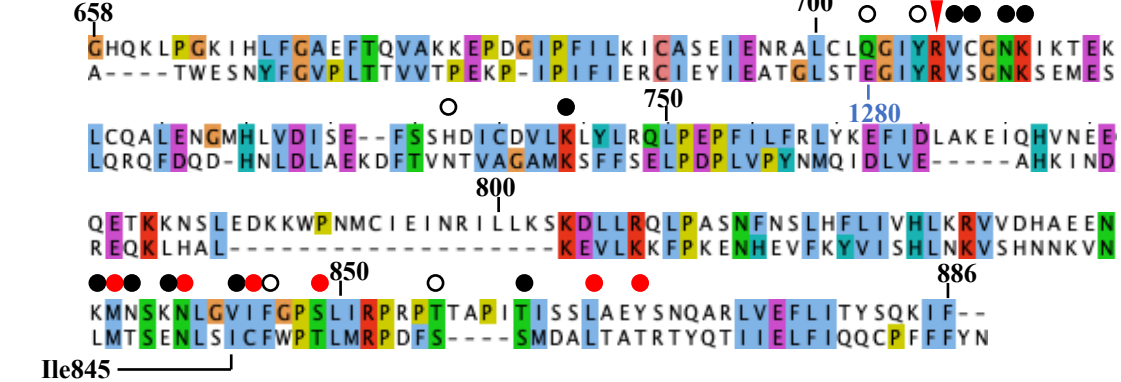

### PARG1##(276aa) 1ow3

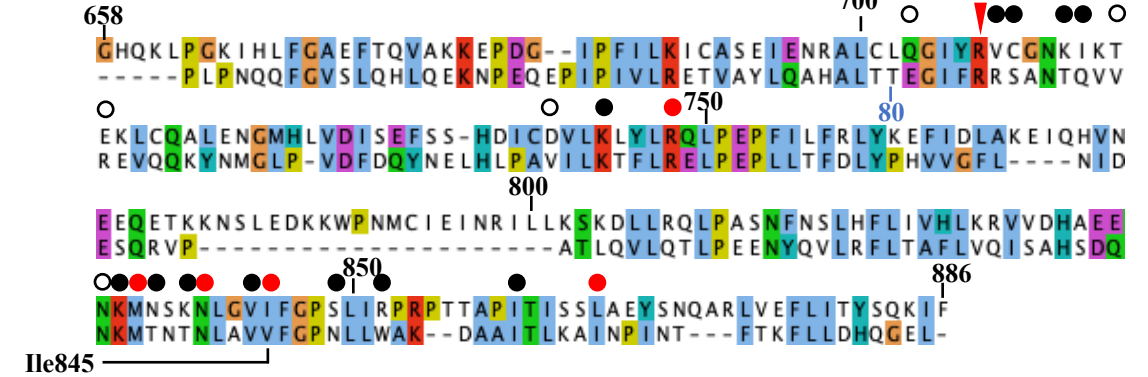

### PARG1## 3msx

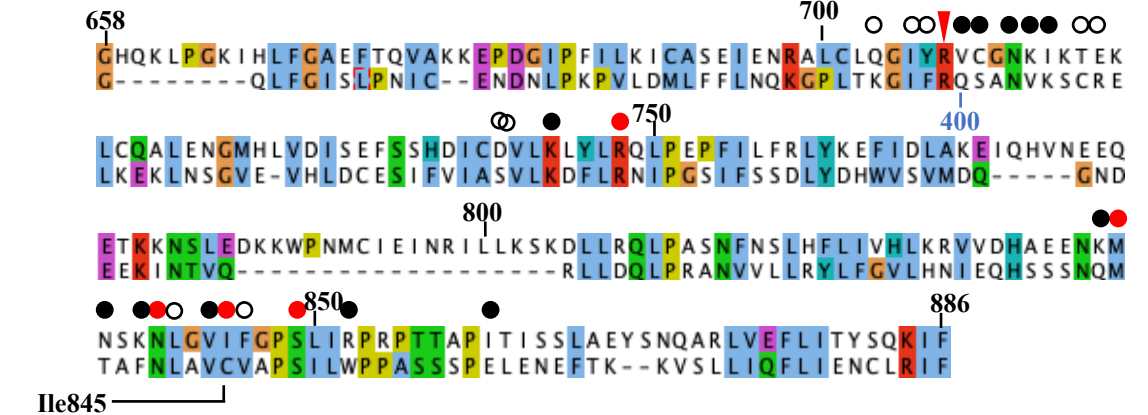

### PARG1## 5irc

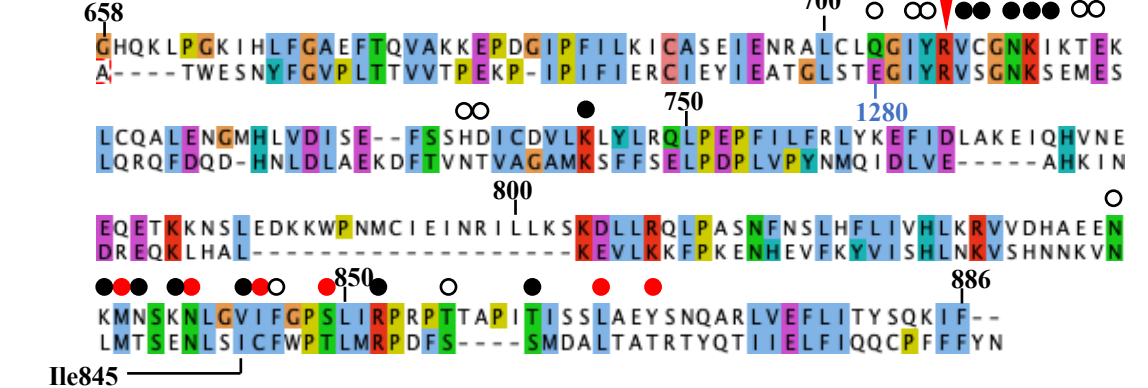

Supplement: S2 Fig — The alignments of GAP region of PARG1 RhoGAP with p50RhoGAP (PDB ID: 1ow3), ArhGAP20 (PDB ID: 3msx), and p190ARhoGAP (PDB ID: 5irc) that form complex with RhoA substrate. Multiple alignments were performed using MAFFT software and color code is based on Clustalσ scheme. The binding free energies were calculated using DrugscorePPI software and the interface residues for RhoA are indicated by circles. The signatures indicate the catalytic arginine finger (red arrowhead), the conserved interface residues (< 4Å) indicated by Amin et al. (2016) matched between PARG1 and each RhoGAP (filled black circle), the interface residues matched between PARG1 and each RhoGAP (black circle), and the conserved interface residues that showed different ϕ and φ angles in 2 μs MD simulation between wild-type PARG1 and missense PARG1 mutants (filled red circle). The Ile845 residue locates as the predicted interface residue for RhoA in PARG1 that is matched to the corresponding interface residue of crystallized RhoGAPs. (PDF) [file pone.0326924.s002.pdf]

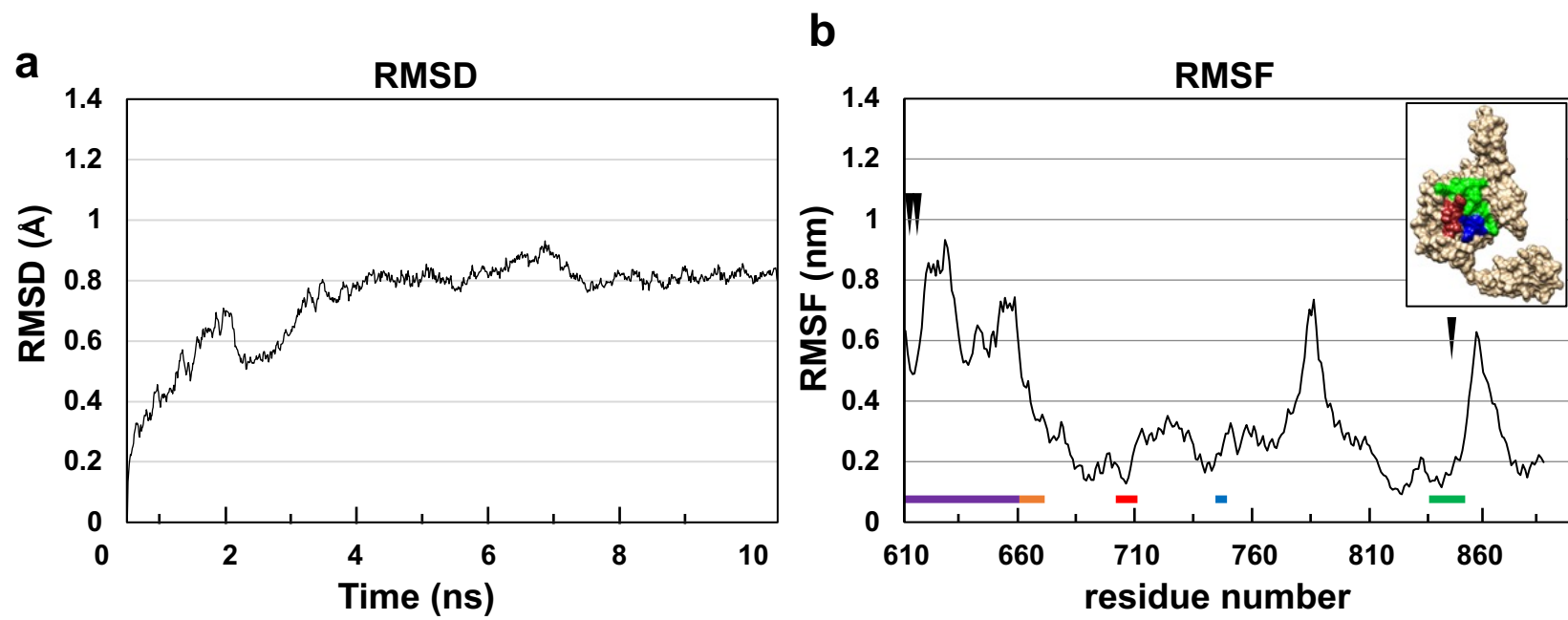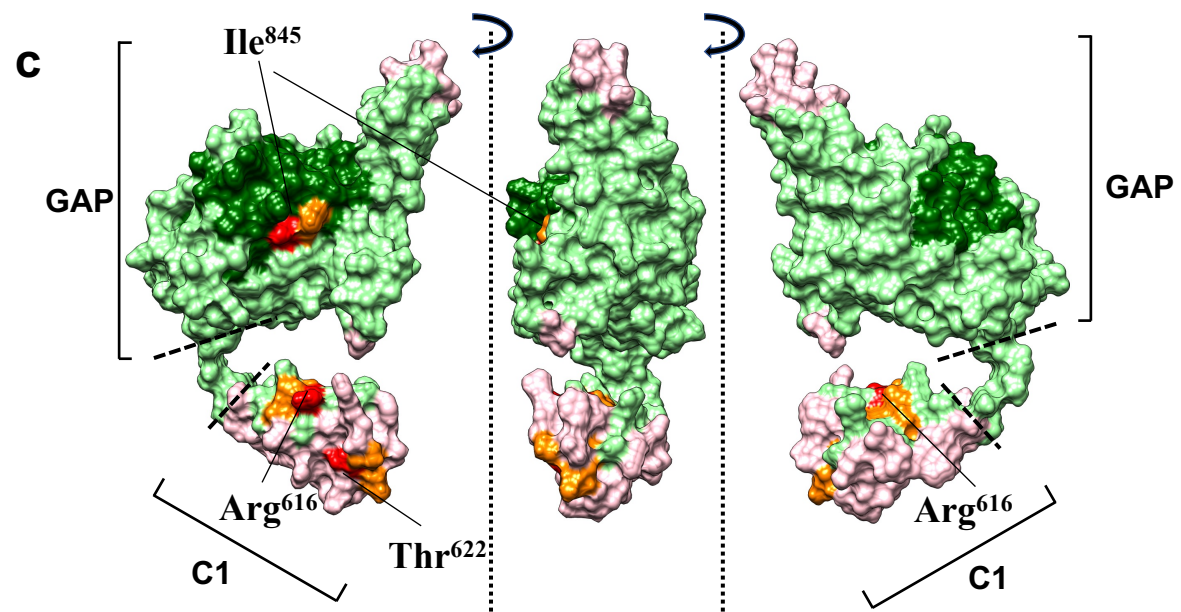

Supplement: S3 Fig — (a) RMSD of modeled C1-RhoGAP domain of WT PARG1 (residue number: 611−886) in 10-ns MD simulation. (b) RMSF of C1-RhoGAP domain (residue number: 611−886). 10-ns MD simulation was conducted and Phe669 and Gly670 played critical roles on the inter-residue interactions with the α3-helix containing the conserved positively charged interface. The conserved interfaces for RhoA interaction are indicated as the follows: purple: C1 domain; orange: loop region; red: catalytic arginine finger; blue: positively charged α-helix; green: C-terminal interface. The conserved residues of PARG1 are mapped on the structural model (inset: brown: arginine finger; blue: positively charged interface; green: C-terminal interface). The arrowheads indicate the mutated sites identified in NSCL/P individuals (R616H, T622M, and I845V). (c) Position of mutated (red) and proximal (orange) residues that locate less than 5Å within the mutated residue (R616H, T622M, I845V) in NSCL/P in PARG1. Flexibility based on RMSF values is indicated (dark green: < 0.2; green: 0.2–0.6; pink > 0.6 in RMSF obtained from 10-ns MD simulation). (PDF) [file pone.0326924.s003.pdf]

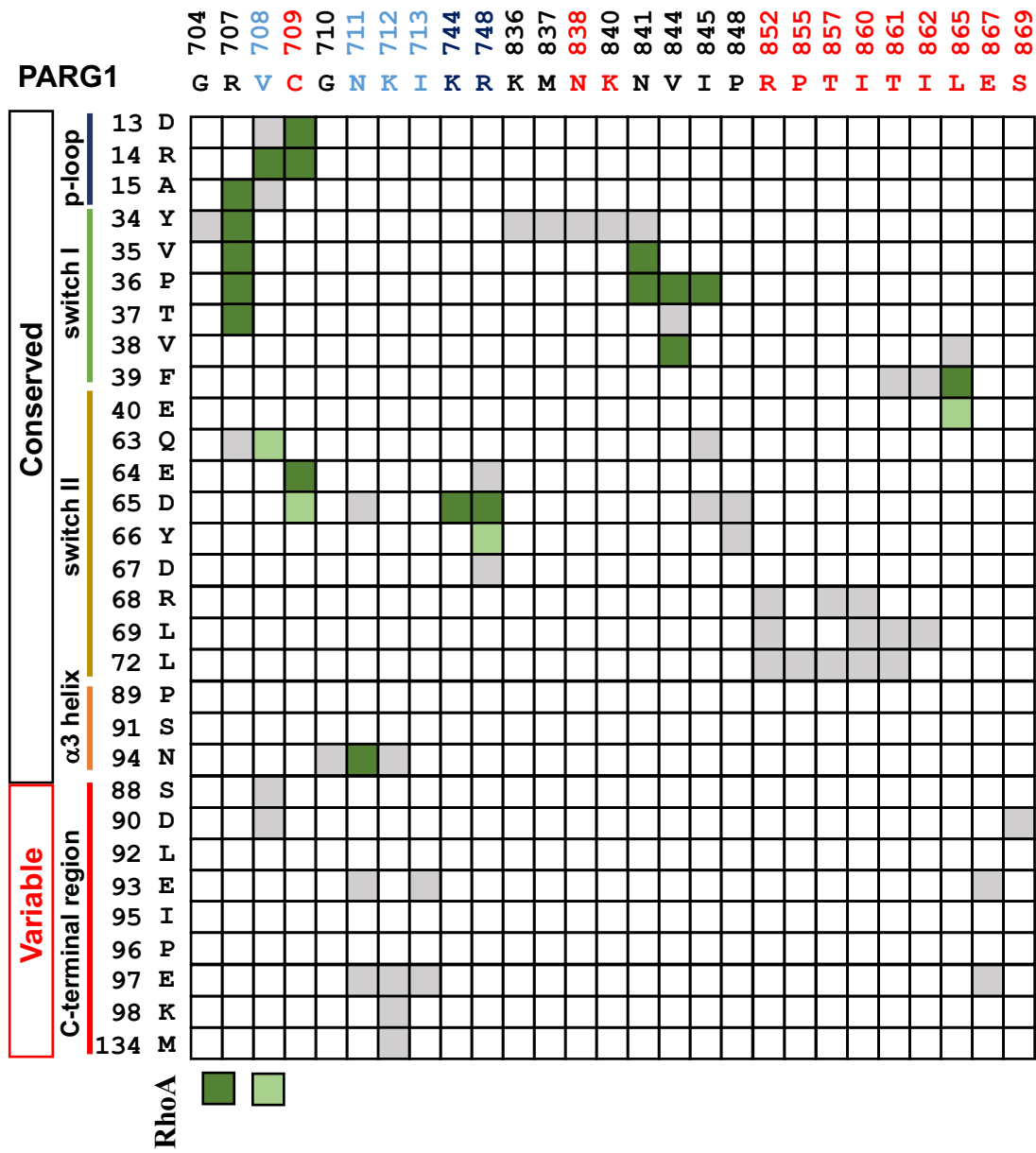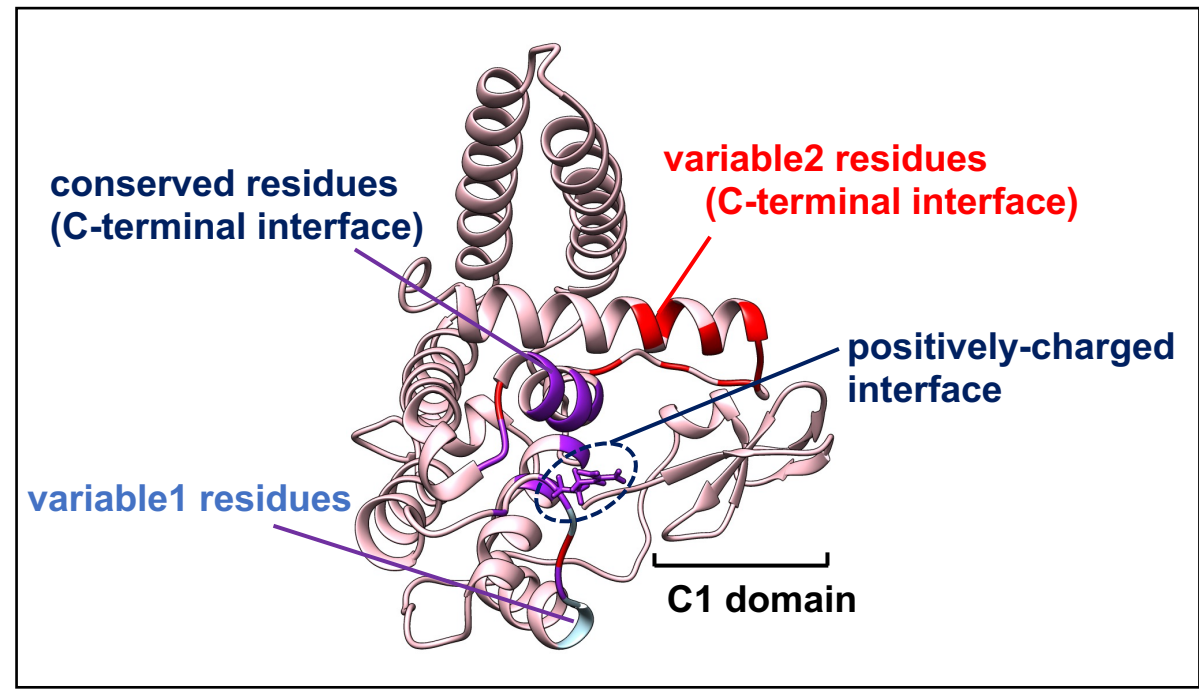

Supplement: S4 Fig — The modeled RhoGAP domain with N-terminal C1 region was subjected to docking analysis using HDOCK in template-free mode. left panel: The interface residues (< 6Å) of WT PARG1 for RhoA binding were shown as overlay of the mapped interaction interface (gray) indicated in Amin et al. (2016) [2]. Conserved, variable residues of the GAP domain, and variable residues (variable1) near the arginine finger (variable2) are indicated as black, red, and blue, respectively. Lys744 and Arg748 residues are indicated as dark blue. The residue-residue contacts in the modeled PARG1 complex are shown in dark or light green color that matched the residues corresponding positions of the eight RhoGAP interface (see Fig 6B in [2]) or not as shown in Amin et al. (2016). right panel: The predicted interface residues of PARG1 for RhoA. The residue color of human PARG1 RhoGAP structure indicates as followed: purple: conserved region, blue: variable region 1, red: variable region 2. dotted circle: positively-charged interface (Lys744 and Arg748). The residues are mapped on the modeled structure according to Amin et al. (2016) [2]. (PDF) [file pone.0326924.s004.pdf]

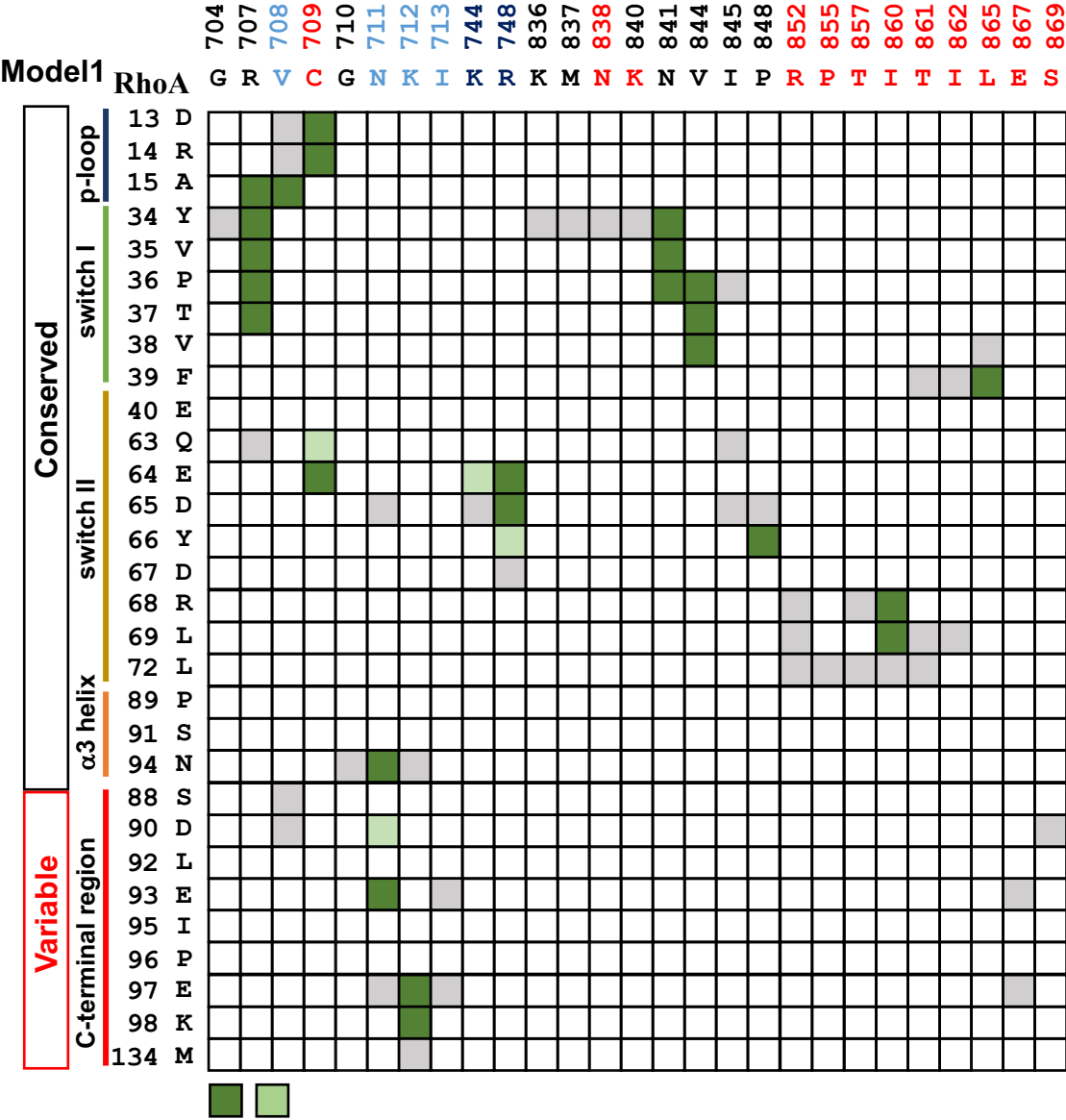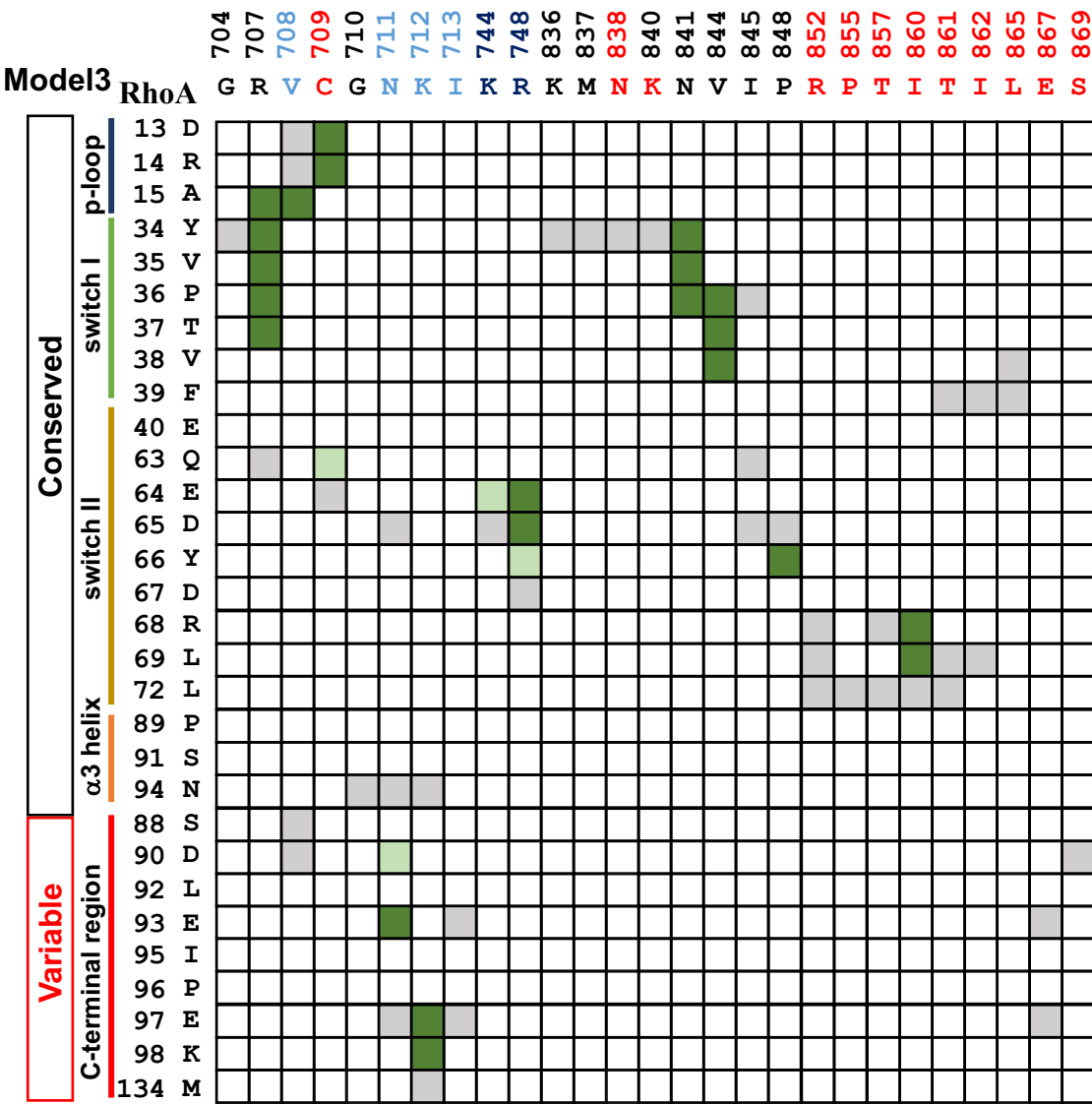

Supplement: S5 Fig — The RhoGAP domains with N-terminal C1 region bound to RhoA were modeled using AlphFold-multimer program. The interface residues (< 6Å) of WT PARG1 for RhoA interaction were shown as overlay of the mapped interaction interface (gray) indicated in Amin et al. (2016) [2]. Complex model 1 and 3 were selected based on RMSD values to the crystallized RhoA complex structures. The residue-residue contacts in the AlphaFold-modeled PARG1 complex are shown in dark or light green color that matched the residues corresponding positions of the eight RhoGAP interface (gray, see Fig 6B in [2]) or not as shown in Amin et al. (2016). (PDF) [file pone.0326924.s005.pdf]

**(a) Cys657**

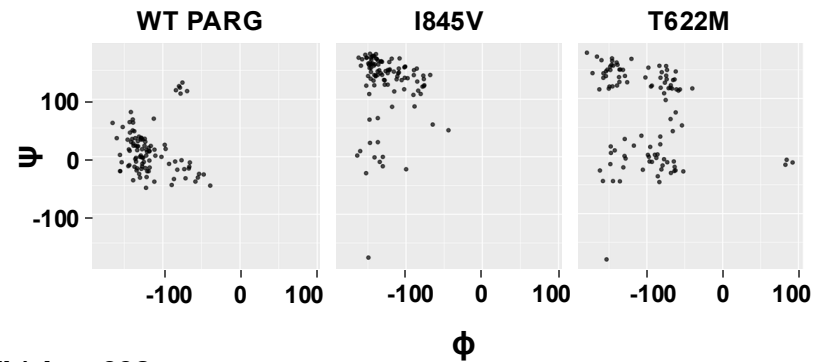

**(b) Leu668**

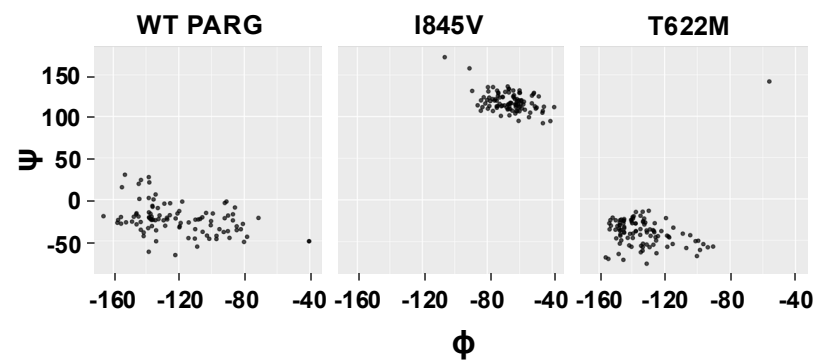

**(c) Phe669**

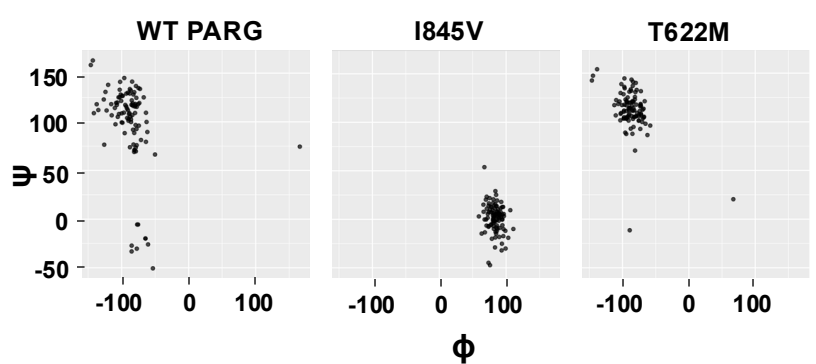

**(d) His659**

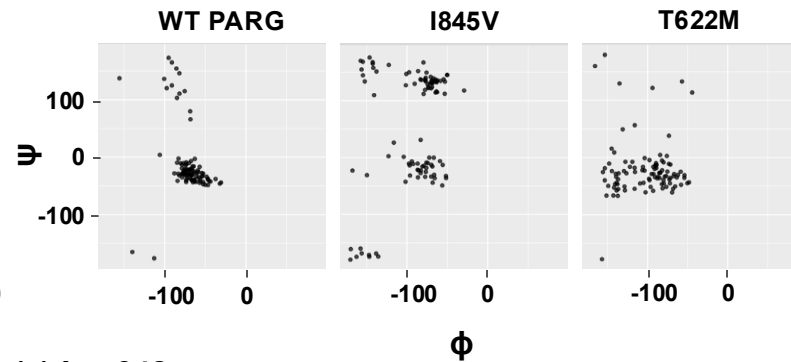

**(e) Lys648**

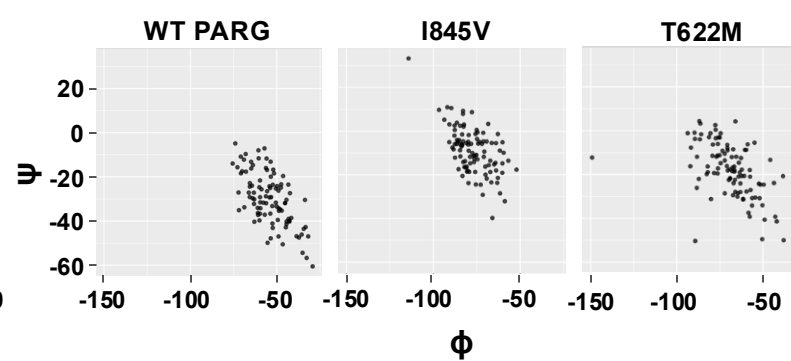

**(f) Thr622**

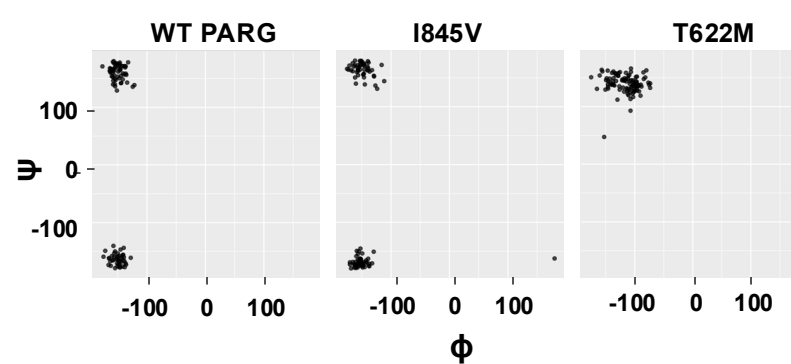

**(g) Lys623**

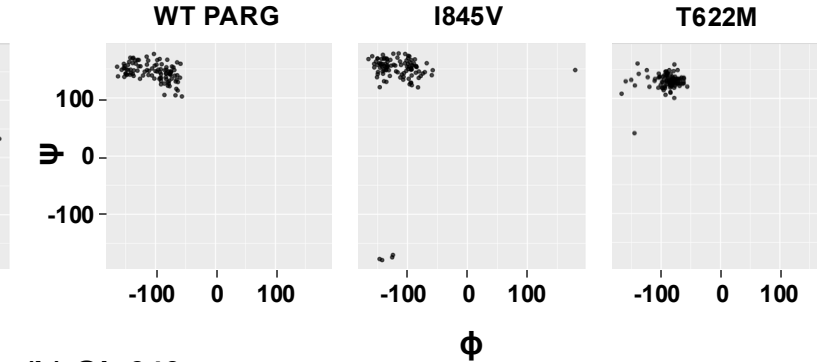

**(h) Glu640**

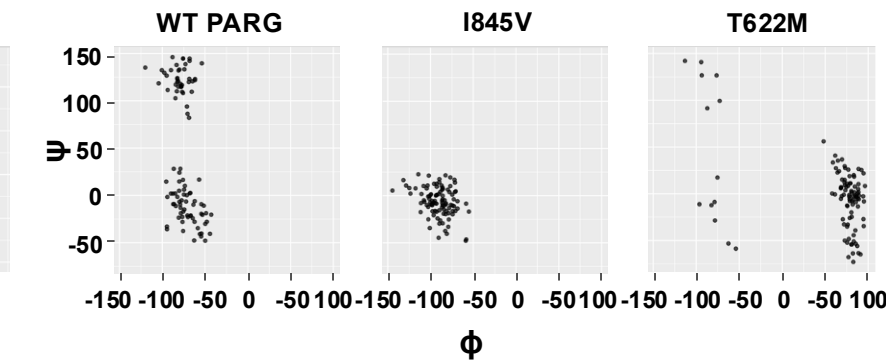

**(i) Leu642**

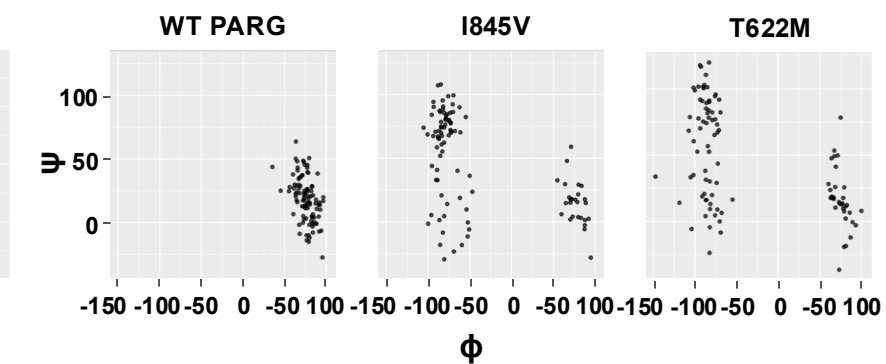

Supplement: S6 Fig — The displayed angles were obtained from MD simulations of C1-RhoGAP/RhoA complexes in 2-μs MD simulation with Amber99SB-ILDN. Cys657 (a), Leu668 (b), and Phe669 (c) residues were selected for WT PARG1-specific motions differed from I845V or T622 mutant complexed with RhoA. His659 (d) and Lys648 (e) residues in the C-terminal C1 domain showed diverse motions among RhoA complexes. Mutated Met622 residue (f) showed angle motions partially distinct from Thr622 residue of wild-type and I845V mutant bound to RhoA. The Lys623 residue (g), the adjacent residues of Met622, also showed T622M mutant-specific motions. The flexibility of an apposed β-sheet was affected by the point mutation, and its N-terminal loop region including Glu640 (h) and Leu642 (i) residues showed different motions from WT PARG1 complex. The Lys648 residue (e) in I845V mutant showed specific motions distinct from WT-RhoA complex, since the residue interacted with RhoA in the mutant complex in 2-μs MD. These data were obtained from the following PARG1 C1-GAP ensembles. WT PARG1, left panel, I845V, Middle panel, T622M, right panel. (PDF) [file pone.0326924.s006.pdf]

WTPARG1

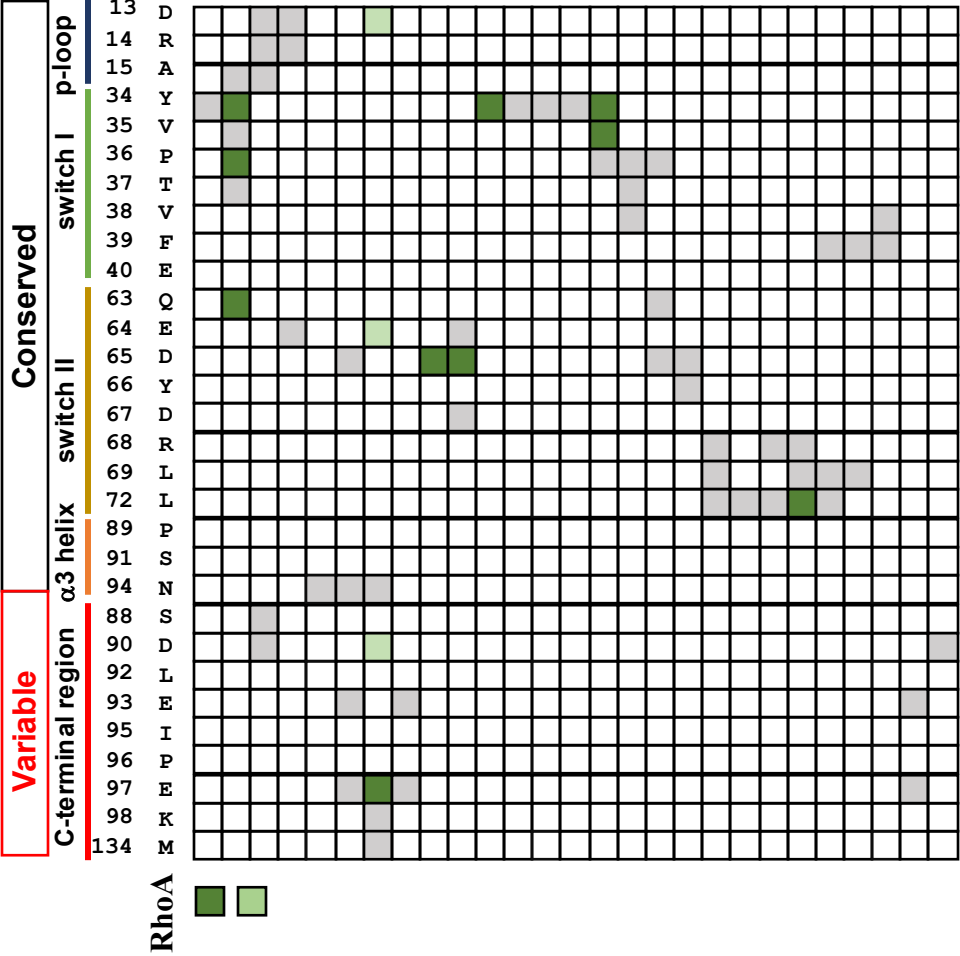

I845V

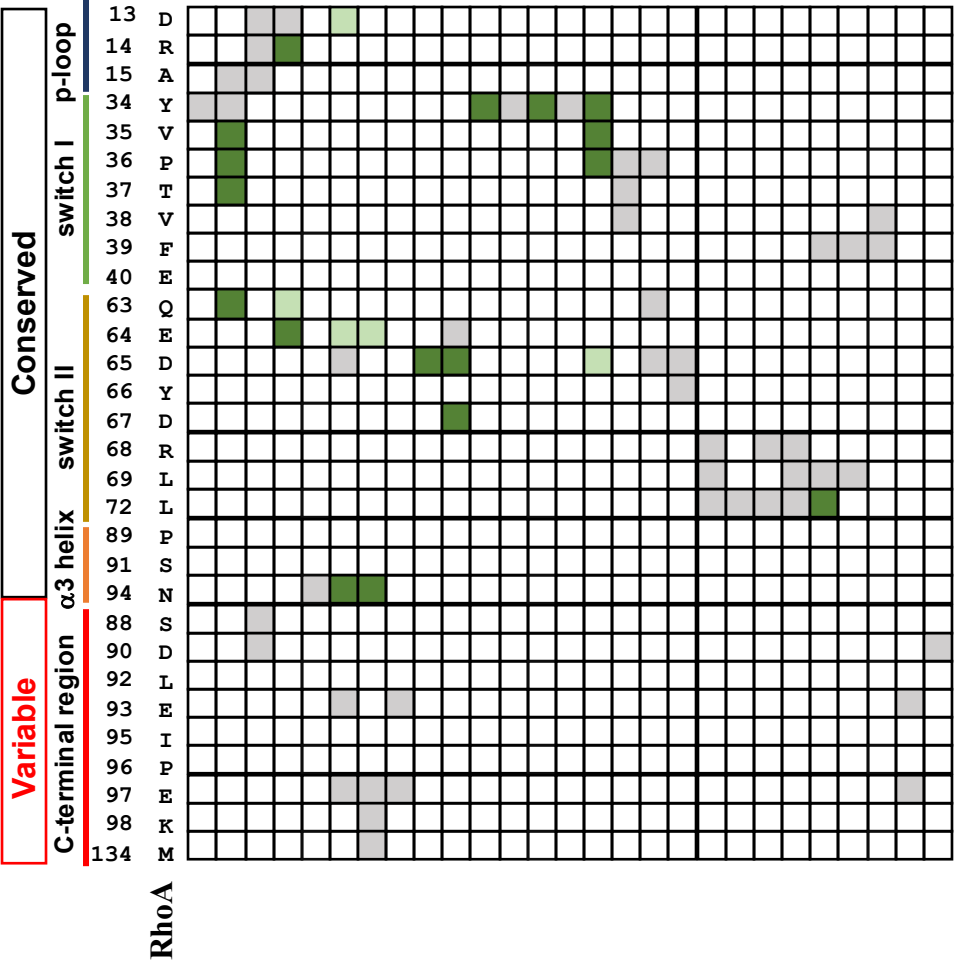

T622M

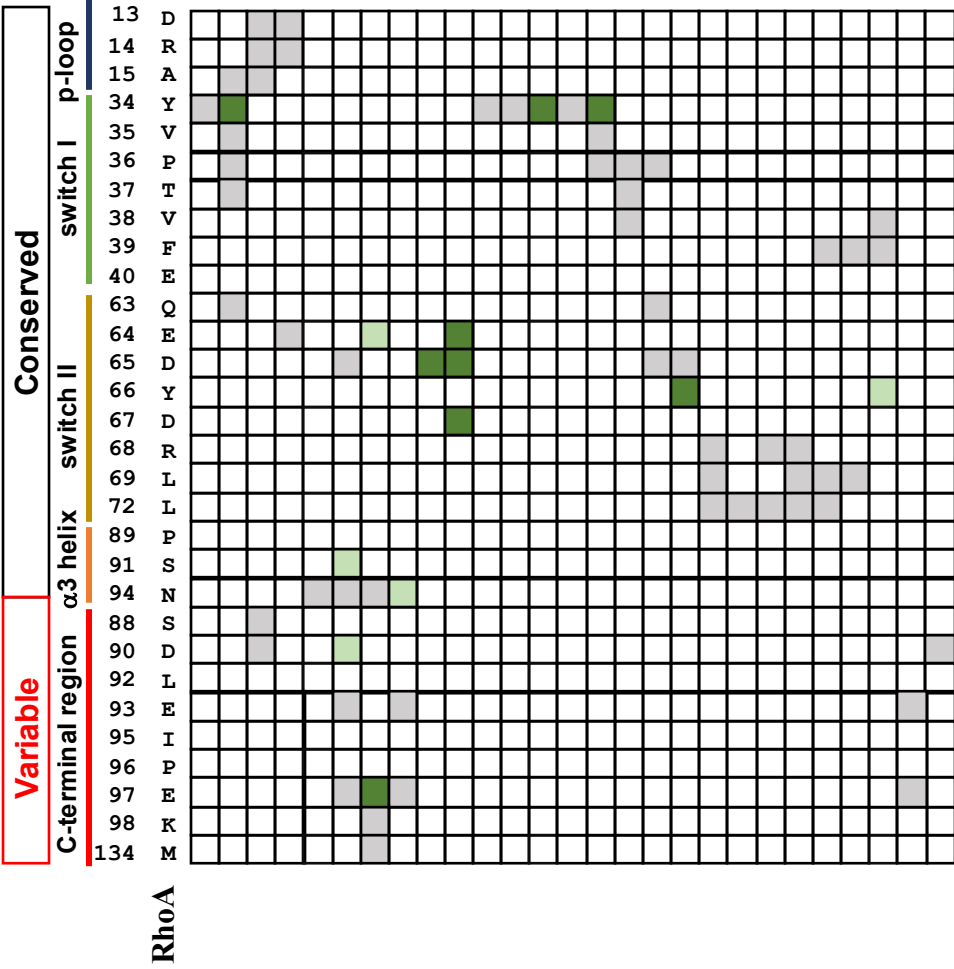

Supplement: S7 Fig — The distribution of interface residues of modeled RhoGAP domains with N-terminal C1 region obtained by 2 μs-MD simulations are mapped: The hydrogen-bonds of interface residues in WT PARG1 or mutant PARG1 proteins and RhoA were shown in dark or light green color that matched the residues corresponding positions of the eight RhoGAP interface (see Fig 6B in [2]) or not as shown in Amin et al. (2016), based on overlay of the mapped interaction interface (gray) indicated in Amin et al. (2016) [2]. Conserved, variable residues of the GAP domain, and variable residues (variable1) near the arginine finger (variable2) in the RhoGAP domain are indicated as black, red, and blue, respectively. The pairwise residue matched or not matched to the corresponding positions of contact residues of the crystallized RhoGAPs (Fig 6B in [2]) are selected, based on the periods forming a residue pair more than 2 ns (0.1%: dark green) and 20 ns (1%: light green), respectively. (PDF) [file pone.0326924.s007.pdf]

(a) Ile845

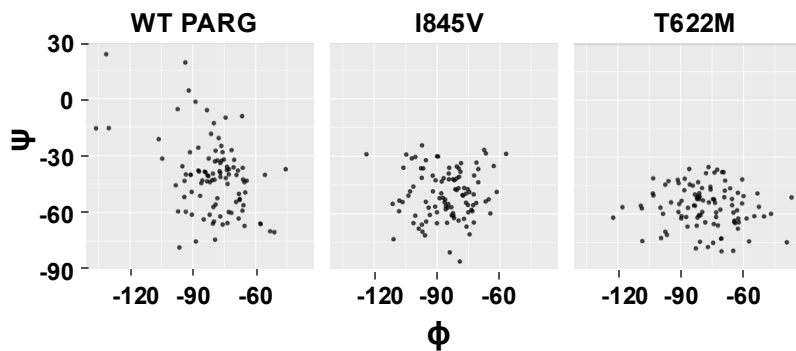

(d) Ile862

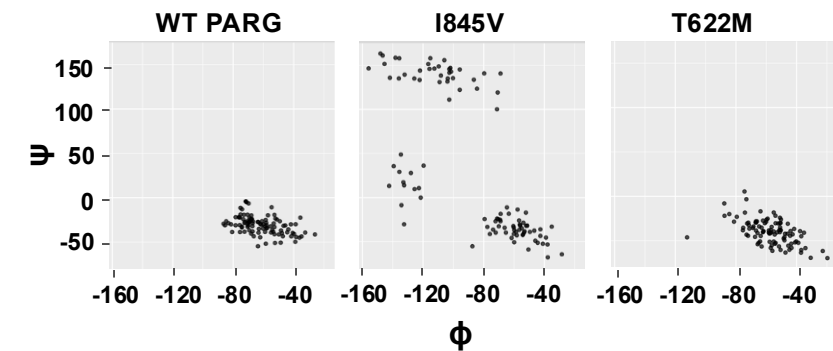

(g) Arg748

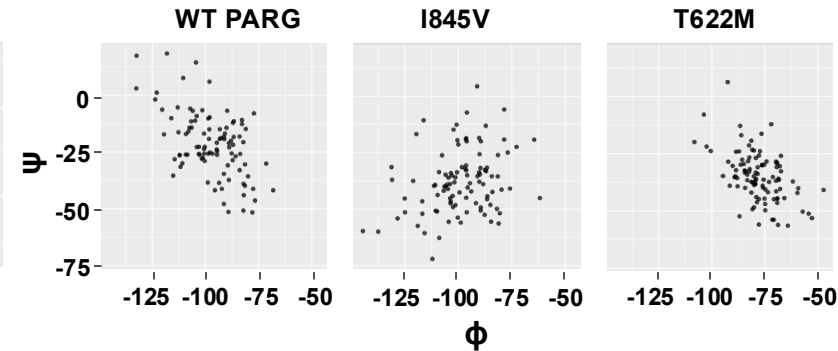

(b) Asn841

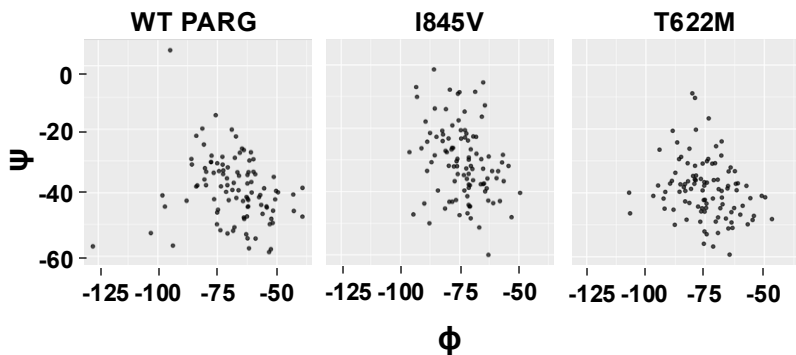

(e) Leu865

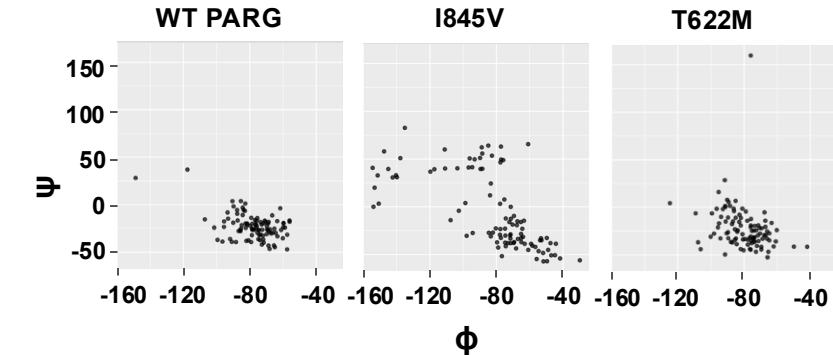

(h) Gln749

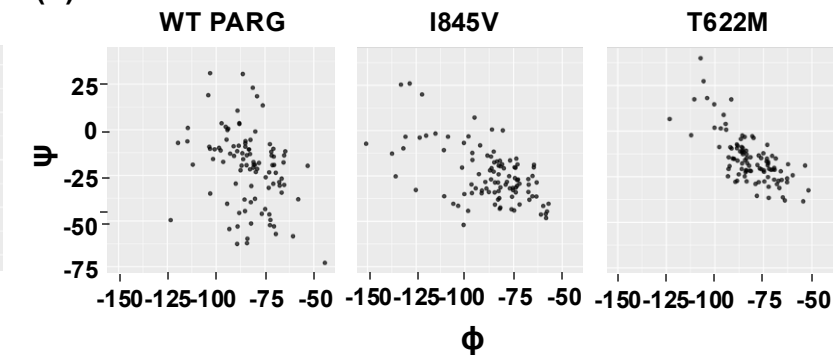

(c) Val844

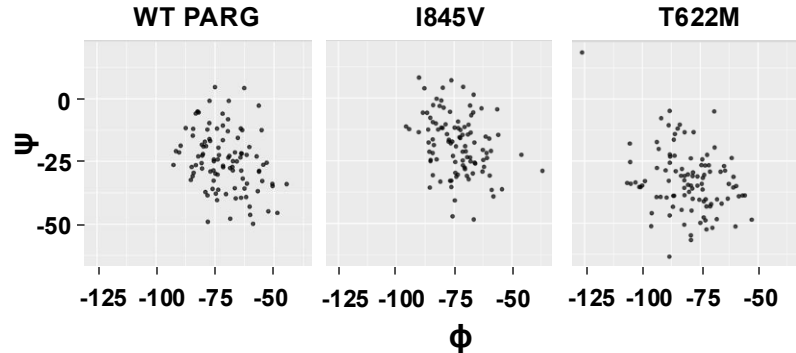

(f) Glu867

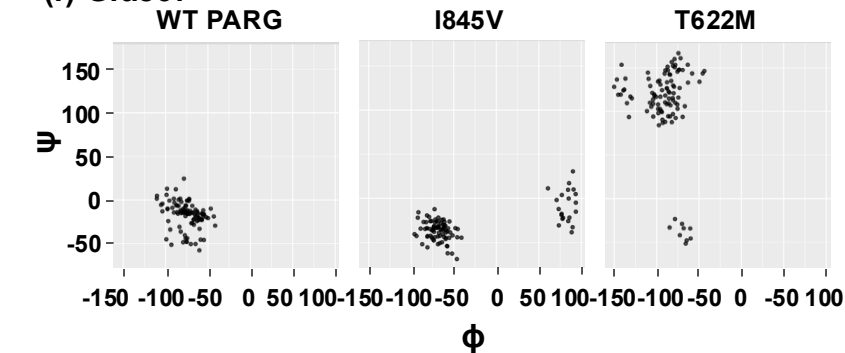

Supplement: S8 Fig — The displayed angles were obtained from MD simulations of WT or mutant PARG1 C1-RhoGAP domains in 2-μs MD trajectories with Amber99SB-ILDN. It is noticeable that mutated residue Val845 (a) in the complex showed motions distinct from Ile845 residue in WTPARG1 or T622M mutant bound to RhoA. The ϕ and φ angles of the adjacent conserved Arn841 (b) and Val844 (c) residues and Ile862 (d) and Leu865 (e) residues in the α10-helix tended to show I845V-specific distortion in RhoA complex in 2-μs MD simulation. In T622M mutant, Glu867 residue in the α10-helix showed distinct motions compared to other RhoA complexes. Arg748 (g) and Gln749 (h) in α4-helix residues were selected for WT PARG1 complex-specific motion angles differed from that of I845V or T622 mutant bound to RhoA. WT PARG1, left panel, I845V, middle panel, T622M, right panel. (PDF) [file pone.0326924.s008.pdf]

(a) Arg852

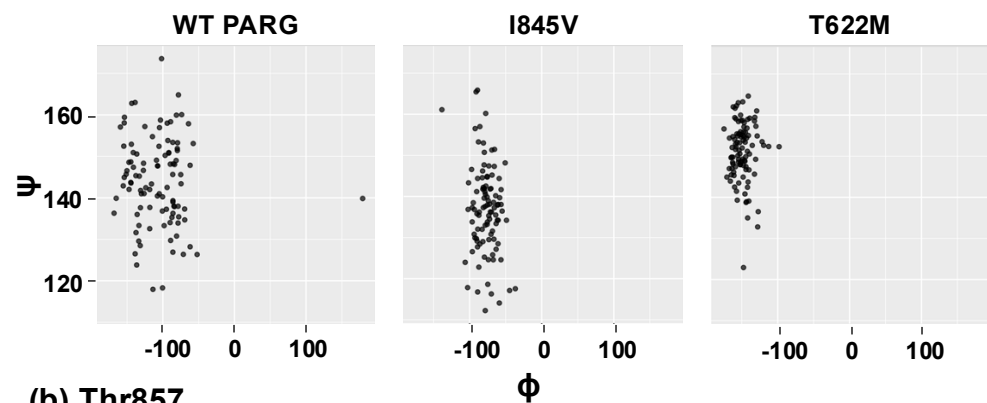

(b) Thr857

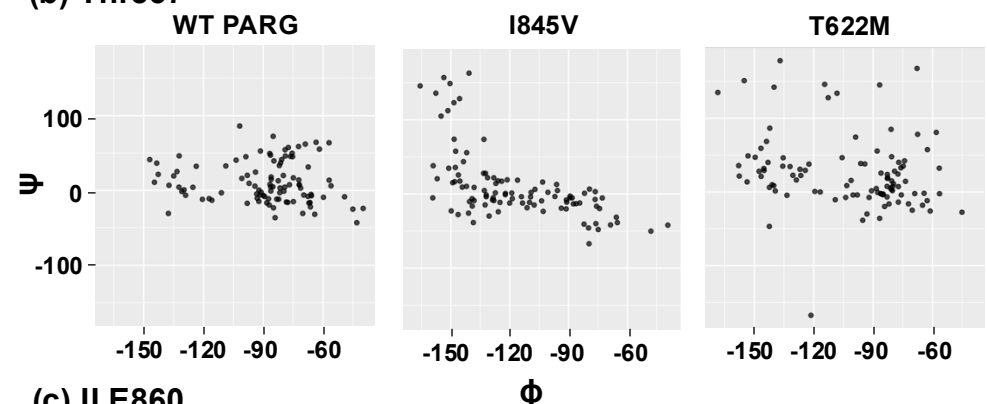

(c) ILE860

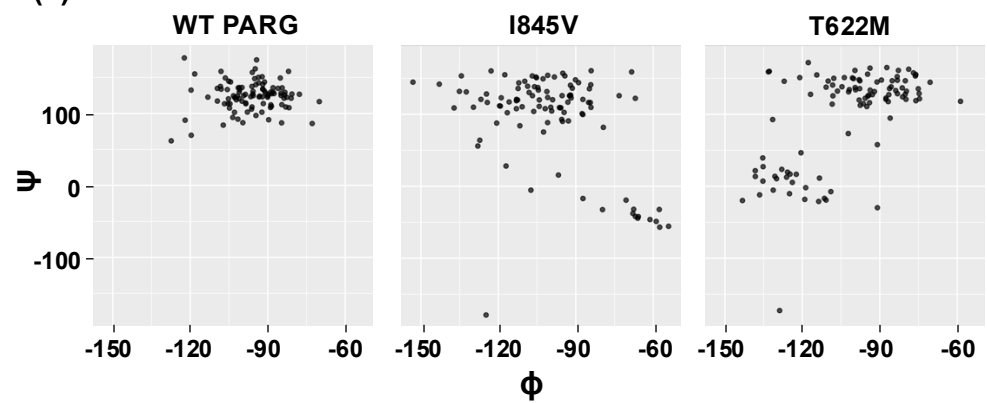

(d) Arg861

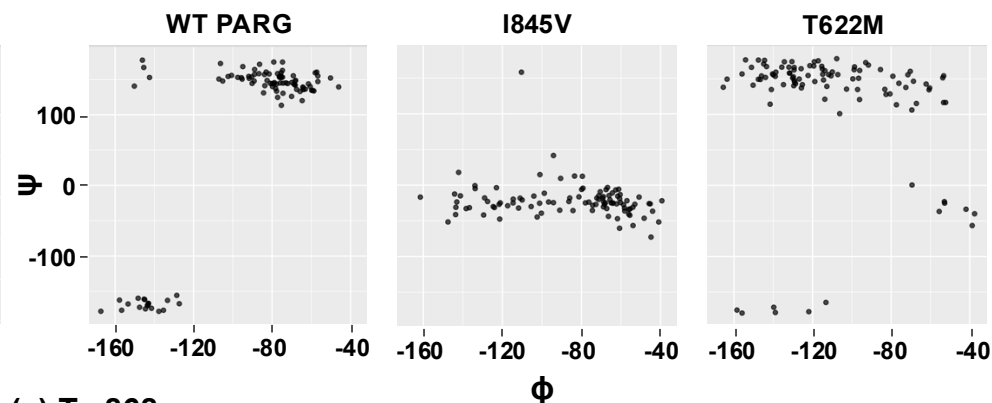

(e) Tyr868

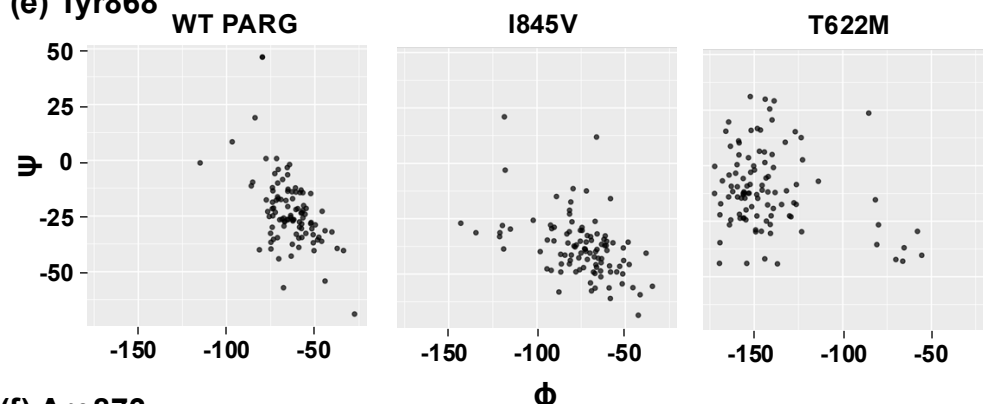

(f) Arg873

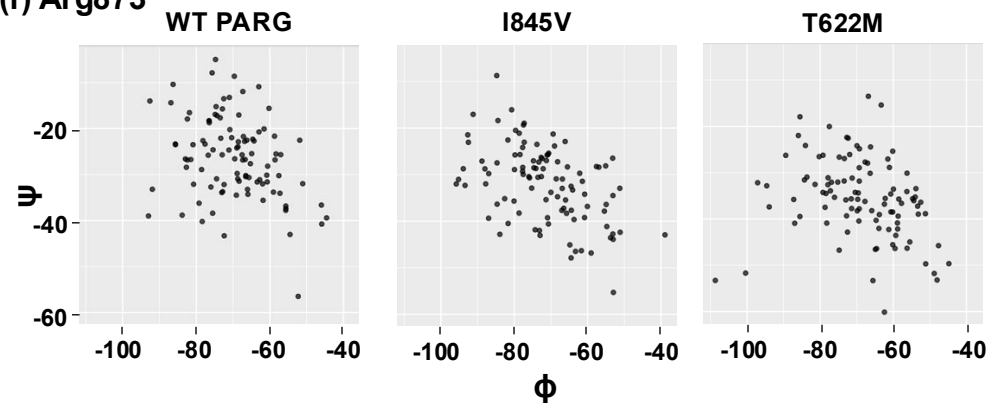

Supplement: S9 Fig — The displayed angles were obtained from 2 μs-MD simulations of WT or mutant PARG1 C1-RhoGAP domains. It is noticeable that Arg852 (a), Thr857 (b), Ile860 (c), Arg861 (d), Tyr868 (e), and Arg873 (f) residues in the α10-helix and its N-terminal loop region of WT PARG1 complex showed specific motions distinct from PARG1 mutants bound to RhoA. WT PARG1, left panel, I845V, middle panel, T622M, right panel. (PDF) [file pone.0326924.s009.pdf]

**a** (i) RhoA (WT PARG)

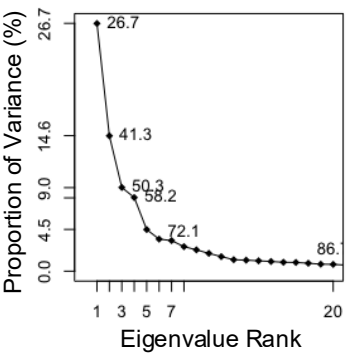

(ii) RhoA (I845V)

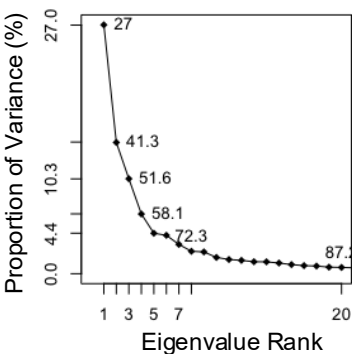

(iii) RhoA (T622M)

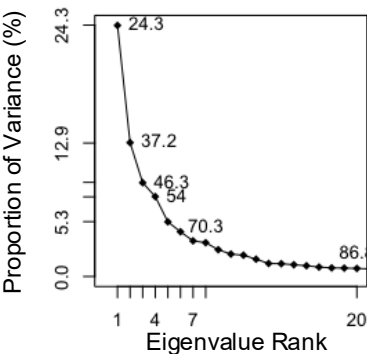

**b** (i) RhoA (WT PARG)

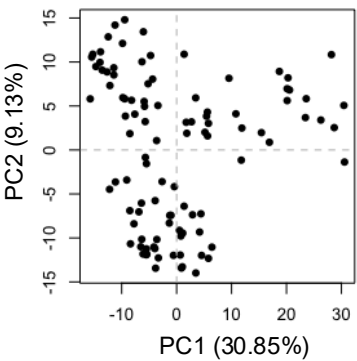

(ii) RhoA (I845V)

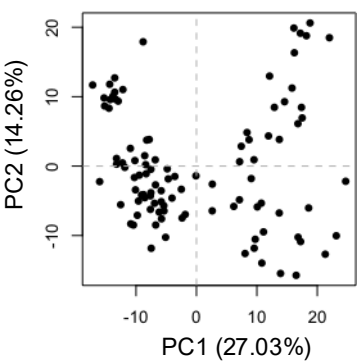

(iii) RhoA (T622M)

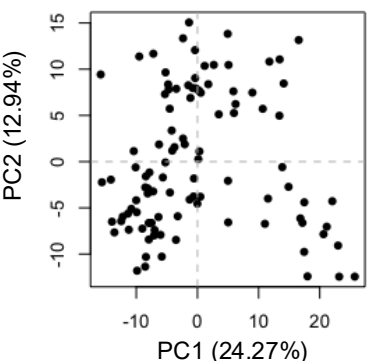

Supplement: S10 Fig — (i) WT PARG1, (ii) I845V, (iii) T622M. The eigenvectors and eigenvalues were calculated by diagonalizing the atomic displacement correlation matrix of Cα atom coordinates from RhoA structures bound to each C1-GAP domain. (a) Screen plot shows the proportion of variance against the eigenvector rank. (b) Projection of trajectory to the planes of the first-two principal components. (PDF) [file pone.0326924.s010.pdf]

WTPARG1

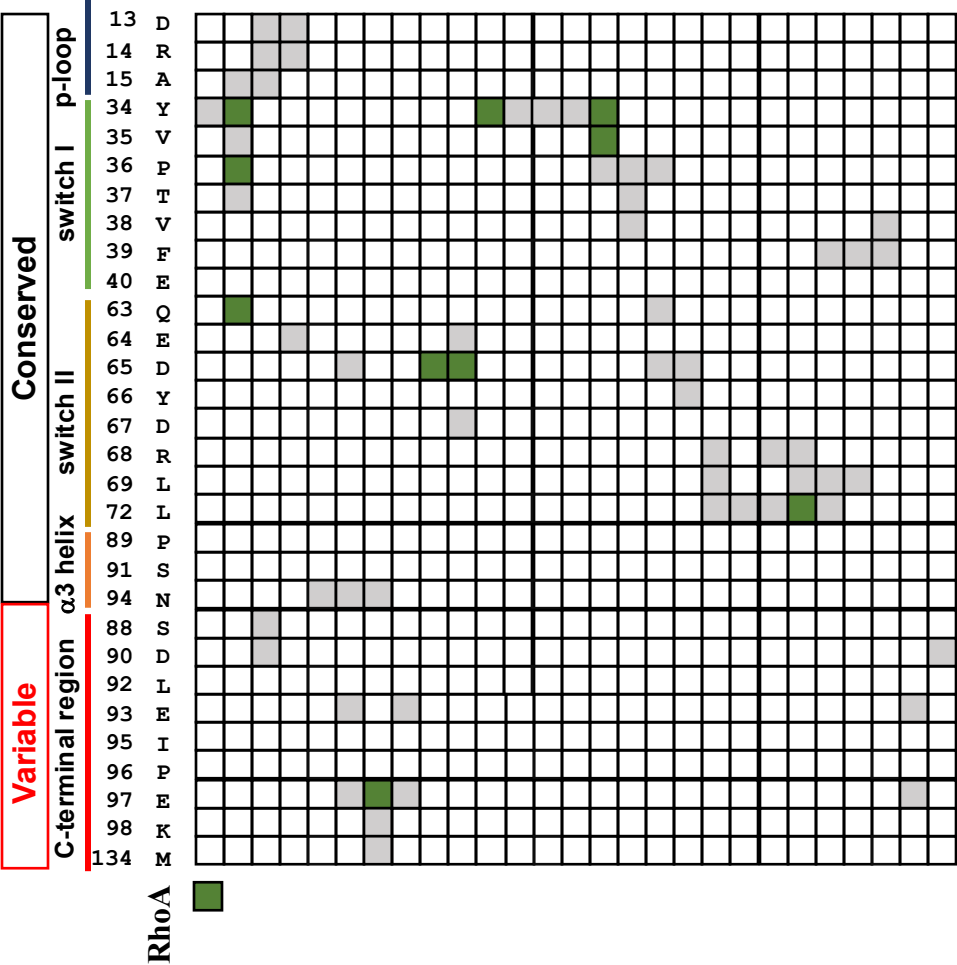

Model1-AF

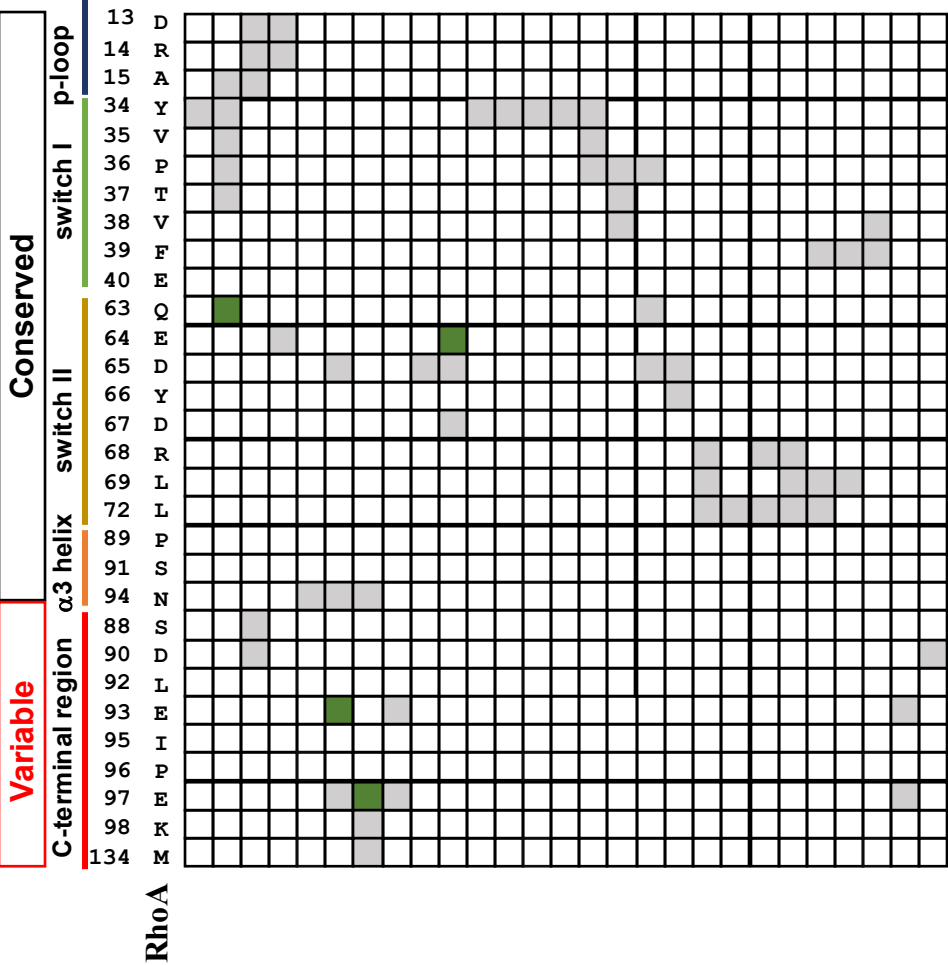

Model3-AF

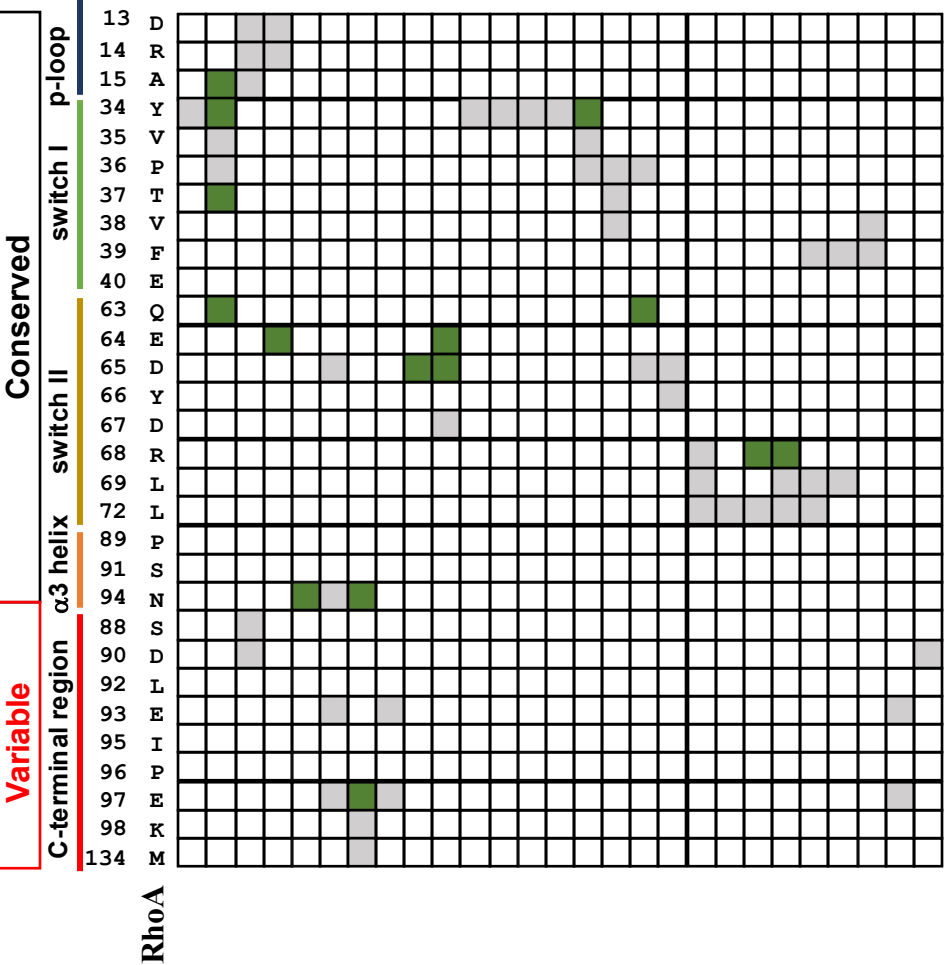

Supplement: S11 Fig — The mapping of interface residues of modeled RhoGAP domains with N-terminal C1 region obtained by each MD simulation: The hydrogen-bonds of interface residues in RhoA-WT PARG1 using HDOCK in 2-μs MD (a) and modeled PARG1 obtained by AlphaFold multimer (b, c) were shown as overlay of the mapped interaction interface (gray) indicated in Amin et al. (2016) [2]. As for AlphaFold-modeled complexes, the profiles of model 1 and 3 were calculated from the trajectories of the stable complexes in 70 ns and 75 ns trajectories in MD simulations, respectively. The residue-residue contacts in the modeled PARG1 complex are shown in dark or light green color that matched to the residues corresponding positions of the eight RhoGAP interface (gray, see Fig 6B in [2]) or not as shown in Amin et al. (2016). (PDF) [file pone.0326924.s011.pdf]
