# Supplementary material for: C1-linker region of PARG1 RhoGAP promotes the catalytic recognition fold of RhoA substrate
Source: PLoS One. 2025 Jul 9;20(7):e0326924. doi: 10.1371/journal.pone.0326924 (PMC12240320; doi:10.1371/journal.pone.0326924)
Supplement: S1 Table — Details of hits for the selected template of human PARG1 GAP domains are indicated. PARG1 (residue number: 671–886): the GAP domain retrieved from InterPro; PARG1#: GAP domain containing the N- and C-terminal loop (residue number: 658–898); PARG1##: GAP domain containing the C1 domain (residue number: 611–886). Prob: Probability of template to be a true positive. E(expect)-value: average hit of false positives. Score: raw score calculated by comparing the amino acid distributions between columns from the query alignment and columns from the template alignment. The probabilities for insertions and deletion at each position in the alignment are taken into accounts as positional specific gap penalties. SS: secondary structure score. Cols: The number of aligned match-match column in the HMM-HMM alignment. Query HMM: Range of query match states aligned. Template HMM: range of template match states aligned and, in parenthesis, total number of template HMM. Id(%): sequence identity. 3cxl.pdb: Structure of chimerin1. 5c2k.pdb: Structure of MgcRacGAP bound to RhoA. 2mbg.pdb: Structure of RalA-binding protein 1. (PDF) [file pone.0326924.s012.pdf]

Target	Template	Prob	E-value	Score	SS	Cols	Query HMM	Template HMM	Id (%)
PARG1 (216aa)	3cxl	99.82	7.60e-18	124.48	19.3	187	3-216	270-459 (463)	34
	5c2k	99.82	9.50e-18	121.68	20.4	182	9-216	357-539 (415)	27
PARG1[#] (241aa)	2mbg	99.81	2.80e-17	118.52	20.5	189	5-236	184-375 (655)	25
PARG1[##] (276aa)	3cxl	99.97	1.90e-28	201.44	20.8	247	2-276	205-375 (463)	33
