# Supplementary material for: C1-linker region of PARG1 RhoGAP promotes the catalytic recognition fold of RhoA substrate
Source: PLoS One. 2025 Jul 9;20(7):e0326924. doi: 10.1371/journal.pone.0326924 (PMC12240320; doi:10.1371/journal.pone.0326924)
Supplement: S2 Table — The selected RhoGAP domains of human PARG1 were evaluated using PROCHECK, ERRAT and ProSA programs. Predicted GAP domain structures retrieved from InterPro search are shown with the template PDB structures in parenthesis. RhoGAP domains containing the N- and C-terminal loop regions (#, residue number: 658–898, 241 amino acids) and the C1 domain (##, residue number: 611–886, 276 amino acids), defined using InterPro software program, were also assessed by the same procedures. (PDF) [file pone.0326924.s013.pdf]

RhoGAP	PARG1		PARG1 #					PARG1 ##				
Modeling Method	SwissM (3cxl)	SwissM (5c2k)	trRosetta (Model1)	trRosetta (Model2)	trRosetta (Model3)	trRosetta (Model4)	trRosetta (Model5)	trRosetta (Model1)	trRosetta (Model2)	trRosetta (Model3)	trRosetta (Model4)	trRosetta (Model5)
Ramachandran Plots (favored)	95.9%	95.8%	100%	99.1%	99.1%	100%	99.5%	99.6%	99.6%	99.2%	99.6%	98.4%
Ramachandran Plots (disallowed)	1.0%	2.1%	0%	0.5%	0%	0%	0%	0.4%	0.0%	0.0%	0.4%	0.4%
ERRAT (overall quality factor)	85.990	94.595	97.36%	95.59%	98.27%	98.28%	97.80%	88.976	93.20	91.188	90.188	89.811
ProSA (Z-score)	-5.97	-5.37	-7.64	-7.56	-7.71	-7.51	-7.75	-7.49	-7.62	-7.57	-7.69	-7.82
