# Supplementary material for: C1-linker region of PARG1 RhoGAP promotes the catalytic recognition fold of RhoA substrate
Source: PLoS One. 2025 Jul 9;20(7):e0326924. doi: 10.1371/journal.pone.0326924 (PMC12240320; doi:10.1371/journal.pone.0326924)
Supplement: Attachment — Submitted filename: Response_To_Reviewers250228.pdf [file pone.0326924.s015.pdf]

Dr. Paul A. Randazzo
Academic Editor
PLoS ONE
Revision of manuscript, submission **ID PONE-D-24-50037 [EMID:1ae5aec5f3027c64]**

Dear Dr. Randazzo,

Please find attached revised manuscript and marked-up copy of the manuscript that highlights changes made to the original version by Kouchi and Kojima entitled "**C1-linker region of PARG1 RhoGAP promotes the catalytic recognition fold of RhoA substrate**", as research article "PLoS ONE". We read carefully your recommendations and the reviewer's criticism and revised the paper accordingly. Here is our point-to-point response to the reviewers' comments and a discussion of all changes made in the revised manuscript.

Editor's comment

(1) The duration of the simulations (100 ns) is important to address. Simulations of at least 2-μsec should be used and these might not be adequate for examining protein complexes that can rearrange on a much longer time scale. The importance of time scale should be discussed in addition to extending the simulations for as much time as reasonably possible.

(2) Using AlphaFold to determine starting structures for MD simulations of the PARG1.

(1) We obtained similar conformational change of the wild-type PARG1-RhoA complex as shown in pattern B (Figure 5) in the original article by conducting another MD-run. We also performed 2-μs scale of MD simulation of the wild-type and I845V PARG1 complexes, and the results were added to the revised manuscript in **Figure 4-9** and **Supplementary Figures 5-7 and 9**. 2. The importance of time scale in the complex was discussed in the **5th paragraph** of **Discussion part** of the revised manuscript. (2) The modeling of AlphaFold was performed but the docking analysis using HDOCK was not consistent with the experimental result that showed specific interaction with RhoA (see Ref. **3** and **10**), MD simulations of the selected RhoA-bound model based on AlphaFold multimer was performed, but stable complex was not obtained more than 50-75 ns through long-ranged simulation analysis: irreversible dissociation patterns of the complex were detected. The details on the complex were added to **Fig. 1h** and in the **result and discussion parts** of the revised manuscript, but the result of MD simulation was not included in the manuscript.

To Reviewer1

To Major Comments

1-1: The manuscript describes the use of traditional computational techniques to model the protein-

protein interactions involving the disease-relevant mutations in the PARG1 RhoGAP domain, however, it is notable the author did not utilize AlphaFold, a highly accurate and widely accepted tool for protein structure prediction, particularly for complex structures. The author could have considered using AlphaFold-Multimer to generate the protein-protein complex models, especially for validating the interactions between PARG1 and its partners.
(Answer) The corresponding region of PARG1 was structurally modeled using AlphaFold software. Cdc42(PDB code: 5c2j) was candidate as substrates for both the AlphaFold-modeled RhoGAP retrieved from InterPro and C1-RhoGAP domains using the HDOCK template-free mode. However, the RhoA-bound forms was previously reported in mono-specificity for RhoA of PARG1 in several papers (Ref. 3, 10). Therefore, RhoA-bound form was also selected using AlphaFold multimer (ColabFold) (Ref. 29,30) and the selected model showing minimum value of RMSD with analogy to the crystallized RhoGAP-RhoA complex (PDB ID: 5c2k) was further analyzed for MD simulation. RhoA structure was predicted based on 2-185 amino acids, corresponding to the used coding region of RhoA as the structure, *i.e.* PDB ID: 5c2k, for docking.

1-2: This would not only have strengthened their findings but also added confidence to their molecular dynamics simulations, as AlphaFold-generated structures tend to serve as better starting points for downstream simulations due to their higher accuracy.
(Answer) Structure of modeled C1-RhoGAP bound to RhoA using AlphaFold multimer (ColabFold) was selected by comparing MgcRacGAP complex structure (ePDB ID: 5c2k). Several analyses on the selected AlphaFold-based structural model did not show any stable motions (50-75ns periods) as the RhoA-bound complex for long simulation period and low levels of dynamics of the C1 domain were detected. Therefore, the long-ranged conformational analysis of the modeled complex was not conducted. Details in the model was mainly described in the **Fig. 1h**, **Result section** and the **2nd paragraph** of the **Discussion part**.

2-1: The manuscript reports performing MD simulations for 100ns, which may be considered relatively short for accurately capturing the full scope of protein dynamics and complex interactions, particularly for systems involving protein-protein interactions or allosteric effects. In many cases, microsecond-scale simulations provide a more comprehensive view of the system's behavior, allowing for a deeper understanding of long-range conformational changes, transient interactions and more stable equilibrium states.
(Answer) Motion property of the C1-GAP domain complex of wild-type PARG1 and its mutant effects in the GAP region on the interaction dynamics of RhoA proteins were especially important issues, therefore the modeled complexes were analyzed for 2-μs MD simulation, using Desmond Molecular Dynamics system. The long-range conformational changes of wild-type PARG1 complex were similar

to the dynamics detected in 100ns-MD simulation, as categorized by "pattern B" in the **Figure 5**. N-terminal positively-charged residues (Lys^{614}, Arg^{616}, Lys^{617}, and Arg^{619} residues) in the C1 domain formed hydrogen bond interactions with Glu^{93} and Glu^{97} residues in the RhoA substrate. In contrast, the corresponding N-terminal C1 domain (Phe^{615} and Lys^{617} residues) in I845V formed stable hydrogen bond interactions with more C-terminal region (Met^{134} and Glu^{137} residues) of RhoA protein. Glu^{639} and Arg^{647}-Lys^{648} residues in the C1 domain also mediated the interaction with Lys^{135} and Glu^{143}/Glu^{93} residues, respectively. Principal component analysis revealed that this C1 domain-mediated interaction caused wide range of flexibility in the N-terminal portion of RhoA, in contrast to the motion of wild-type PARG1 complex as shown in **Fig. 8h**. The catalytic Arg^{707} residue of I845V mutant complex was very close to the several residues located in switch I and II regions (Pro^{31}, Glu^{32}, Val^{35}, Thr^{37}, Asp^{59}, Gln^{63} residues) more than 20ns periods in transient state. In addition, Lys^{714} residue in the C-terminal catalytic loop of the mutant complex formed hydrogen bond with Asp^{100} residue of RhoA (see **Figure S4**). The summary of the hydrogen interaction network is indicated for wild-type PARG1 complex in **Fig. 6b, c in the revised figure,** and the motion dynamics of I845V mutant complex was described in the **Discussion part**.

2-2: Protein-protein interactions and allosteric effects, for instance, may not fully manifest over just 100ns, as significant networks can take longer to develop. Rare events, such as the binding/unbinding if ligands or conformational transitions, might require longer simulation times to be adequately sampled.
(Answer) The conformation of the RhoA complex indicated in **Fig. 5a-B** was not one of the rare events in that the C1 domain formed the interface to RhoA substrate. Another trajectory with similar structural motion of the complex with **Fig. 5a-B** has also been obtained using the same condition of 100-ns MD simulation.

3-1: While the manuscript provides a solid computational analysis of the structural changes caused by T622M and I845V mutations, it lacks a detailed discussion on how these findings correlate with published biological data. The connection between these computational observations and known experimental studies on the effects of similar mutations in RhoGAP proteins (or homologous proteins) is not adequately addressed.
(Answer) Phenotype of I845V mutation was reported by the following papers, Leslie et al. (2012) Birth Defects Research. Part A, Clinical and Molecular Teratology and Tang et al. (2020) *BioMed Res. International*. As shown in the paper, details in physiological or biochemical effects of the mutation were not described. YFP-tagged PARG1 was reported to be poorly expressed in HEK293 cells (Anderson EL and Hamann MJ, *Biochem. J.* 441: 869-871, 2012) and difficulties in cellular studies of PARG1 have been suggested. All the references on the variants are included in the **revised version**.

3-2: Mutations in the RhoGAP domain are known to affect the regulation of RhoA GTPases, a key player in cellular processes such as migration and cytoskeletal organization. Including references to experimental studies linking RhoGAP mutations to cellular dysfunction would strengthen the manuscript and provide biological context.

(Answer) The reported experimental studies linking RhoGAP mutations were added to the **3rd paragraph** of **Discussion part** with additional references. All the listed references are referred to HGMD database (http://www.hgmd.org), and pathological missense mutations within the GAP region associated with cytoskeletal disorganization have been reported in the following RhoGAP proteins: ArhGAP24 (R142C, Q158R), Oligophrenin1(OPHN1) (G412D, M461V, Q529R), N-chimerin1(CHN1) (E313K).

4: The manuscript introduces an intriguing link between PARG1 RhoGAP mutations and NSCL/P, but the connection remains speculative. The discussion could benefit from more focus on the developmental biology of cleft lip and palate and how RhoA signaling is involved in craniofacial development. Altered cellular dynamics resulting from disrupted RhoA regulation are likely contributors to developmental disorders, but this connection is not sufficiently explored.

(Answer) Physiological roles of RhoGTPase signaling including RhoA, associated with cytoskeletal reorganization in craniofacial development, were added to the **1st paragraph** of **Discussion part** with references. Pathological RhoA activation in cleft cells from NSCL/P patients are known to cause reduced cell motility with Cdc42 activation, but coordinated activation of several RhoGTPases in neural crest cells and craniofacial mesenchyme during embryonic development has been experimentally validated using conditional knockout or dominant negative Rho kinase mutant models.

5: MMPBSA analysis is a useful tool for estimating the binding free energy differences between mutant and wild-type protein-protein complexes. In the context of the T622M and I845V mutations, this method could help quantify how these mutations affect the interaction stability between PARG1 and RhoGAP, potentially linking changes in interaction energy to disease-causing effects.

(Answer) MMPBSA analysis was performed based on the representative trajectories considering 2D-RMSD analysis and these data were added to the manuscript as **Table 1**. Wild-type PARG1-RhoA complex in pattern B (**Fig. 5**) showed lower binding free energy (-56.02 kcal/mol in No.1; -79.64 kcal/mol in No.2 in **Fig. 4**) than those of T622M (-33.40 kcal/mol in No.1; -48.47 kcal/mol in No.2) and I845V complexes (-48.54 kcal/mol in No.1; -38.11 kcal/mol in No.2), indicating that the C1 domain promoted the stable interaction of the GAP domain with RhoA protein. The mode of stable interaction or irreversible interaction during 100ns MD simulations did not reflect the values of binding free energies of mutant PARG1-RhoA complexes obtained by MMPBSA, suggesting that the

conformation of RhoGAP domain in the complex, in addition to its affinity to the RhoA substrate, may regulate their stability of RhoA interaction. This conclusion was also supported by the similar value of binding free energies of wild-type PARG1-RhoA complex (-31.97 kcal/mol) in pattern A (**Fig. 5**) to those of mutant PARG1 complexes.

6-1: The RMSD plots presented in the manuscript lack clarity regarding their generation, raising questions about the apparent discontinuities. These breaks could result from improper post-processing of trajectory data, potentially due to periodic boundary conditions (PBC) not being correctly accounted for during the RMSD calculations. Proper recentering and imaging should be performed to ensure a continuous and accurate trajectory representation.
(Answer) RMSD values of the RhoA-dissociated complex was corrected in **Fig. 4** based on the conditions in the simulation box with 8 Å in minimum distance between the box and the protein. The data on long-ranged MD simulation analysis was also added to the **Fig. 4**.

6-2: Furthermore, the figure legends are overly descriptive but fail to provide sufficient explanation for critical features in the plots. For instance, the annotations “1”, “2”, and “3” in the RMSD plots are introduced without a clear description of what they signify. The author should revise the legends to concisely explain these elements and ensure the figures are intuitive and informative.
(Answer) The MD trajectory was selected based on summation of the duration (ns) of the stable complex and the trajectories showing minimum period of dissociated forms was presented as the representative trajectories.

To minor comments
Several sections in the manuscript contain repetitive information, especially regarding the interactions of specific residues with RhoA and their role in protein stability. This redundancy can detract from the clarity of the manuscript.
(Answer) The redundancy for the description on the interface residues and stability was avoided by changing the presentation of figures and description in the revised manuscript.

The figures are well-designed and aid in understanding of the computational results. However, some figure legends are repetitive and could be streamlined to highlight the most important findings.
(Answer) The main points for the analysis were concisely presented as the revised figure, and summarized schemes of the motion analysis was added as the **Figure 9**.

Y-axis label units are missing in Fig. 8a.
(Answer) Y-axis unit was labeled in **Fig. 8a** in the revised manuscript.

To Reviewer2

To Major Comments

1: The major shortcoming of the paper is that all of the data is simulated, not experimentally validated. For example. Demonstrating that the C1-GAP construct of PARG1 (residue 611-886) exhibits greater catalytic activity than the GAP domain with N-and C-terminal ends extended (residues 658-898) would be appropriate, as the author argues that the C1 domain modulates GAP activity through interdomain (GAP) and substrate (RhoA) interactions. Furthermore, testing the activities of I845V and T622M C1-GAP constructs would corroborate the author's proposal that their catalytic efficiencies are decreased compared to WT enzyme. In lieu of these experiments, the author should strongly reiterate in the abstract and discussion that their findings are solely based on molecular modeling, docking, and dynamics, and that these findings need to be experimentally validated in the future.

(Answer) The aim performing molecular modeling and molecular dynamics analysis, but not experiments, was added to **Abstracts** and the **2nd and last paragraph** of **Discussion part**.

2: Although touched upon briefly, a thorough analysis of the R616H mutation was not performed. Could the author comment on why this was not done, or instead, add these analyses to the manuscript?

(Answer) R616H mutation was reported in 55 individuals with NSCL/P cases from the Philippines as described in Table1 of Leslie's paper (published in 2012, Ref 4), but 34 control samples include this variant as a rare variant category, which is in contrast to the T622M and I845V NSCL/P cases only, categorized as very rare variants. The Arg606 locates the 1st β-sheet in the predicted structure of the C1 domain (Fig. 3) and both of the Arg and His residues possess similar amphiphilic properties with the long side chains. Therefore, The R616H mutation was not selected for the analysis and structural role of Thr^{622} residue in the C1 domain was focused in this study for the interest in MD simulation. Actually, considering the motions of the C1 domain of the RhoA-bound WT-PARG1 (**Fig. 5a**) or I645V and T622M complexes (**Fig. 9, see discussion**), it is difficult to add to the study and discuss the mutational effects on the RhoA interactions in the conformational dynamics (Arg^{606} residue was not involved in the interface for RhoA in the I845V mutant complex in 2-μs MD simulation. The Arg^{606} residue mediated hydrogen bond interactions with Glu^{637} or Glu^{639} residues in the middle of the C1 domain in the both complexes).

To General Comments

1: "Hydrophilic" seems to sometimes be used instead of "hydrophobic", please check each usage on a case-by-case basis and ensure that the correct term is used. For example, many residues within "hydrophilic" interphase appear to be those that are classically considered to be hydrophobic.

(Answer) The part of "Hydrophilic" interface in the text was corrected to the "<u>C-terminal</u> interface"

in the text of the revised manuscript, considering the position in the GAP domain.

2: Figures have so many labels that it is difficult to distinguish individual features. Consider removing ones that are unnecessary to convey the point (e.g., many amino acid labels in figures 1, 2, 3, 5, and 6).

(Answer) The repetitive labels in the figure1, 2, 5 and 6 were removed to describe the results concisely.

3: Statements saying "conserved" and "matched" are not always clear., please use in a more quantitative sense. What does "highly conserved" and "well-matched" mean? Similarly, regions of the GAP are referred to as "conserved variable" or "variable conserved" regions, which is not intuitive to the reader as these have opposing meanings.

(Answer) The statement "conserved" means conserved as the interface position, based on the category of the interface residues for RhoGTPases in RhoGAP domains shown in **Fig. S4** and supplementary Figures1 and 2 of Amin's paper (Ref 2). "highly conserved" means almost identical in the corresponding position in the sequence. The sentence including these expressions were checked and, for example, "conserved variable/"variable conserved" was corrected in pp.15 and 38 of the revised manuscript.

4: Might the patterns of C1 domain interactions depicted in Fig. 5A (arising from MD simulations) be altered in the full-length protein? i.e., if the F-BAR and linker region to C1 domain were present, would it restrict the movement and cause a preference for either pattern? In other words, how biologically relevant are the two patterns that are proposed?

(Answer) The F-BAR (ZPH: ZK669.1a and PARG1 homology) region of Spv1 (*C.elegans* homolog) directly interacts with C25D7.7 protein, a *C. elegans* Rap2a homolog, which belongs to small GTPase protein (Myagmar BE *et al. Biochem. Biophys. Res. Commun.* **329**: 1046, 2005). PARG1-Rasip1 complex has been known to interact with GTP-bound form of Rap proteins as a separate module for translocation to the plasma membrane (see **Ref.44**). Therefore, similar F-BAR functions, independent of the C1-GAP domain, are deduced to be conserved among mouse and C. elegans PARG1 as the separated domain. However, the corresponding regions depicted 25.8% sequence homology between the species and it remains obscure that the tertiary complexes are similar or not. Therefore, the discussion for these issues was not included in the revised manuscript in view of the structural property of F-BAR domain and biological significance. Two patterns in the WT PARG1-RhoA complexes are indicated from the aspects of their reversible interaction and stable formation in the dynamics.

5. Might the mutational effects of T622M not stem from changes in the dynamics of the $\alpha4$ and $\alpha9$ helices as indicated on page 13, but instead simply from steric clashes caused by the methionine that

prevent either pattern A or B (Fig. 5A) from forming? T622M seems to be localized near the interfaces in both patterns, particularly pattern B (although it is hard to tell from the figure).

(Answer) The backbone and of β-sheet containing Thr^{622} residue in WTPARG1 and Met622 in the missense mutant and ϕ-φ angles of these residues were not significantly changed in tertiary structures during 100-ns and 2-µs simulations. There were not differences in the distances of the region consist of Arg^{619}-Arg^{625} residues (7 amino acid, see **Fig. 3a**) to the opposed β-sheet (Glu628-Val631) or to adjacent hairpin structure (Glu^{637}-Val^{644} residues) between trajectories of WTPARG1(**Fig. 4a-ii**) and T622M mutant (**Fig. 4d-i**). This means conformation of the C1 region is relatively similar and stable between these two trajectories (see also **Fig. 7a (i) and (iv)**). Therefore, the probability that steric clashes caused by the Met^{626} residue derived from the C1 motion prevent A or B from forming may be very low. The distance profiles of Arg619-Arg625 in MD trajectory performed using MDAnalysis (Michaud-Agrawal et al. *J. Comput. Chem*. **32**: 2319, 2011) are shown below.

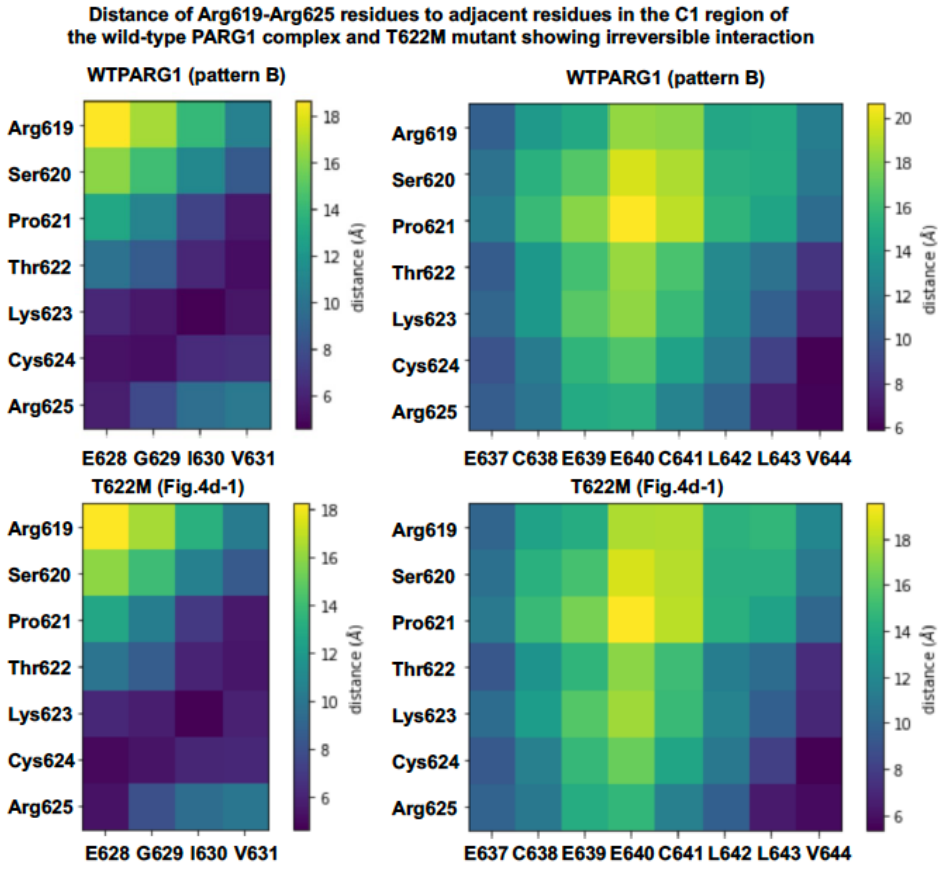

The possibility of steric clashes was also addressed by the positional Thr^{622} effect on the RhoA affinity using alanine mutational analysis of the C1 domain of WT PARG1 complex (pattern B) in MM-PBSA analysis. The result was described in the **6th paragraph** in the **Discussion part**.

6: It would be appropriated to add a cartoon summary figure showing the localization of the C1 domain

relative to GAP domain and bound RhoA substrate in all MD simulations (i.e., patterns A and B in WT, and mobile in mutant PARG1). Furthermore, the summary figure should succinctly explain the major effects caused by each mutation (for I845V, destabilization of N841 contacts with concomitant movement of C1 domain leading to loss of interactions/affinity to RhoA switches I and II; and for T622M, due to altered interface of C1 domain to RhoA and subsequent movements of C1-linker and GAP helices α4 and α9, leading to loss of RhoA switch I contacts). This would make the overarching findings of the study much easier to follow.

(Answer) The cartoon summary figure was added as a new figure, **Figure 9** in the revised manuscript. The figures contain the representative structures of the complex models obtained in each MD simulations, and adequately summarized the positions of the C1 domain as superimposed images to discern the motion dynamics of WT PARG1 as pattern A vs B, and mutant PARG1 complexes. See also the comments of **No.2-1 to reviewer1**.

Introduction

・line1: as written, RhoGEFs appear as though they regulate GTP hydrolysis rather than exchange. Please correct.

(Answer) The corresponding sentence was corrected by adding the explanation of RhoGEFs and RhoGDIs.

Results

・Page 10, R748 does not appear to be conserved in 5irc (p190ARhoGAP) but is stated to be a critical residue for the RhoA interface?

(Answer) Most of the RhoGAPs (53/57) contain the Arg residues in the corresponding site of the α_5 in the GAP domain, and the exceptional cases in the meaning of variable residues are p190RhoGAP (A and B), GMIP, and ArhGAP19 based on the multiple alignments of them in **Ref. 2**. Its putative critical role categorized as "conserved" interface for RhoGTPase is shown in **Fig. 6B and Fig.7 in Ref.2** from the structural aspect. The sentence in p.11 was corrected in the **Result** section.

・Page 10, "The calculation using Drugscore^{PPI} showed that predicted interface residues of PARG1 GAP domain containing the C1-domain were well-matched to those of the other RhoGAP complexes bound to RhoA, compared to the RhoA bound GAP domain with the N- and C-terminal regions." There doesn't appear to be much of a difference between PARG1# and PARG1## in Fig. S2?

(Answer) Fig. S2 was corrected to **Fig. S1** in the corresponding sentence in p.11 in the revised manuscript.

・Page 10, this is the first mention of residue R616 other than in supplemental figures, please introduce

this mutated residue sooner (e.g., in Introduction).

(Answer) The description on R616H variant was added to the 2nd **paragraph** of **Introduction** part in the revised manuscript.

・Page 10, "point mutation at Thr622 affected a more flexible region than the Arg616 residue in the C1-GAP domain during the 10-ns MD simulation (Fig. 1d, Fig. 3b). "Perhaps this is meant to cite Fig. 1F rather than 1D?

(Answer) The reference was corrected from Fig. 1d to **Fig. 1f**.

・Page 11, how is the theoretical binding affinity of I845V calculated to be 10-fold lower than WT when the binding free energies are unchanged? As for T622M, Fig. 4 suggests that the periods in which RhoA is dissociated from the GAP are increased for both mutants, which would perhaps suggest a lower affinity; is the 100ns simulation representative of apparent affinity?

(Answer) The theoretical binding affinity was based on the equation defined by PRODIGY software in the initial complex. It did not reflect the binding free energies during the structural ensembles, therefore the binding free energies were calculated using gmx_MM-PBSA. The binding free energies of the representative trajectories calculated by MMPBSA showed that the T622M mutant in irreversible interaction showed higher value (-33.40kcal/mol) than the stable T622M-RhoA complex (-48.54 kcal/mol), suggesting that the lower affinity caused the RhoA dissociation during 100-ns simulation. These values are reasonable compared to those of the Graf1-RhoGTPase complex, but weaker than the reported experimental values obtained from ITC experiment (Ref.57). In case of I845V mutant, the higher affinity did not cause the stable association with RhoA among its trajectories (**see the comment No.5 to reviewer 1**), indicating that structural integrity for RhoA, in addition to the affinity, may be involved as the determinants for the complex stability. Apparently, the involvement of the C1 domain interface for RhoA in wild-type PARG1 promoted its stable complex formation (the binding free energies: -79.64 kcal/mol in No.2).

・Page 12, "The flexibility of the His659 and Lys648 residues reflected the stability of the RhoA-bound complex, as a wide range of ϕ-φ angles were detected in the stable complex formed during the 100-ns simulation. (Fig. S5b, c, g)." Please clarify, how does the flexibility of these residues indicate stability of the GAP:RhoA complex?

(Answer) Comparing the ϕ-φ angles of WT PARG1 complexes that form in stable and unstable states (Fig. 5 (ii), wider ranges of ϕ angles of His659-Phe669 were detected as seen in **Fig. S5 (ii-b,c,e).** The stable complexes of WT PARG1 and I845V mutant tend to show a wide range of distribution of His659, Leu668, and Phe669, even in 2μs MD simulation. Limited range of ϕ-φ angles in Lys648 residue was detected in WT PARG1(**Fig. 5ii**) and it may be important for conformational changes in the C-terminal

region of the C1 domain in the RhoA complex (see **Fig. 5a**, upper superimposed panel in pattern B). This part was corrected in **p.15** in the Result section.

・Page 13, “Mutational effects of T622M and I845V were attributed to the C-terminal positively-charged α4-helix interface (Fig. S6b and S6c) and the N- and C-terminal edges of the α9-helix of the GAP (Fig. 6a, Fig. S6d-S6f). “Please highlight these regions in the main Fig. 6A and/or cite the relevant residues in the main text.

(Answer) Indicated structure of WT PARG1-RhoA complex in **Fig. 6A** was changed to the representative complex structure obtained from 2-μs MD simulation, and positions of the positively-charged α_4 helix and N- and C-terminal edges of the α_9-helix (Asn838-Arg852 residues) of the GAP domain were highlighted with side-chain marked with brown and orange, respectively. These regions are also indicated with green and yellow ovals on the ribbon structure.

・Page 14, typo “The β1-sheet of the C2 domain” should say “C1 domain” correct? Similarly, later on the page, “The N-terminal motion of the C2 domain…” should say “C1 domain.”

(Answer) The corresponding sites typed “C2” were corrected to “C1” in the text (**p.11 and p.17** of the revised manuscript.

・Page 14, same sentence: “The β1-sheet of [C1] domain…was closed to the C-terminal α3-helix…” Could the author clarify what “closed” means?

(Answer) “closed” was an error of “close” in the sentence and correct it in the revised form (**p.17**). The β-sheet containing Met^{622} residue is adjacent to the α_3 helix of the GAP domain of dissociated form of the T622M mutant C1-GAP domain.

・Page 15, switch I of RhoA appears to be mobile in one RMSF trace in I845V mutant (Fig. 8A iii), but this isn’t reflected in the principal component analysis (Fig. 8C and 8D)?

(Answer) RMSF trace and its principal component analysis in I845V mutant obtained from 2-μs MD simulation are indicated in the **revised Figure 8e-h**. One RMSF trace in I845V mutant previously showing high values in the C1 domain was derived from the trajectories of stable state of the RhoA complex that showed an irreversible dissociation after the period, therefore, the principal component analysis was not included in the revised manuscript.

Discussion

・Page 16, could the author comment more on the ways in which the AlphaFold model-derived docking was different than reported literatures?

(Answer) Considering the interface residues calculated from binding free energies, Cdc42 was selected

as the structural model obtained from AlphaFold software using HDOCK analysis. This result was not consistent with the biochemical data from ref. 3 and 10. The C1-RhoGAP complex model for RhoA obtained from ColabFold software was selected based on the minimum RMSD value to the crystallized RhoA complex (PDB ID: 5c2k) and shown in **Fig. 1h** in the revised manuscript.

・Page 18, last paragraph: the discussion centered on switch I and how its mobility is necessary for catalysis should be elaborated on (perhaps this could be a part of the summary figure, see General comment #6).

(Answer) The reference 68 presented the evidence of the structural pattern of an alternative inactive conformation of GTP-bound RhoA. Switch I region adopts several distinct conformations, and crystallized form selects the one conformational state from multiple possible states in both GDP and GTP-bound states. The flexibility of the switch I region could be adapted to the complex WT PARG1 model obtained from RMSF and principal component studies. These comments are added to **the last** paragraph of **Discussion** part.

Figures

S1: Please make labeling of PARG1* and PARG1 the same as elsewhere in manuscript (i.e. PARG1$^{\#}$ and PARG1$^{\#\#}$) and make the residue ranges shown for RhoA in panels B and D consistent (The ranges shown for WTPARG1$^{\#\#}$ is truncated at 102 versus 134. Main text (p.9) indicates that RhoA from 5irc is used for this analysis, but legend states 5c2k; please clarify.

(Answer) The structural information for PARG1 was unified by using the following term, PARG1$^{\#}$ and PARG1$^{\#\#}$ for the residue number 658-898 and 611-886, respectively. The residue ranges were adjusted in the data of WT PARG1$^{\#\#}$ in **Fig. S1**. The explanation of PDB code in **b, d** for the bound-RhoA was added to the parenthesis in the figure legend.

S2: Please make labeling of PARG1* and PARG1 the same as elsewhere in manuscript (i.e. PARG1$^{\#}$ and PARG1$^{\#\#}$). Please indicate where residue 845 is located, and add more numbers to each line, "700" is not sufficient for the reader. The circles (filled black, black, and red) are not very easy to interpret in terms of meaning, please re-write legend to explain more clearly (e.g. what is different between filled black and black circles?)

(Answer) The structural information for PARG1 was unified by using the following term, PARG1$^{\#}$ and PARG1$^{\#\#}$ for the residue number 658-898 and 611-886, same as **Fig. S1**. Residue number was also added to the sequence alignment to check the position of residues easily. Filled black is corresponding to the conserved residues among RhoGAP species that is picked up in Amin's paper (see **Ref.2**). Black circle means the conserved interface residues in the crystallized structure and modeled structure. Red circle indicates the critical residue for the complex conformation showing wild-type PARG1-specific

ϕ and φ angles as indicated in **Figure 6a**. These definitions are added to the figure legends in **Fig. S2** in the revised manuscript.

S3B: Please label C1 and GAP domains.
(Answer) C1 and GAP domains were labeled in the **Fig. S3c** in the **revised** Figure.

S4: The left panel is not very intuitive, for example what do the colors on the left-hand side (RhoA) represent? As for the squares, what is the difference between dark and light green? The legend indicated that they are "corresponding positions of the eight RhoGAP interface or not," but what eight interfaces are we referring to? On the right the color coding is not accurate, conserved residues are shown as purple (not black as stated in the legend). Does HsPARG1 refer to human PARG1? Please use consistent nomenclatures.
(Answer) The colors on the left-hand side in RhoA means the specific region such as p-loop and switch I or II, and labeling was added to the part. The categorized residues of RhoA interface were labeled as blue, black, and red colors in the left upper row, and eight RhoGAPs are described in Figure 6B in Amin's paper. The box with gray color indicates the interface residues conserved among the RhoGAPs, and dark green box means the matched interface residue in WT PARG1 RhoGAP with this, while light green box means the residue that is not matched. Please see the part of the **reference 2**. The right color coding in structural image was also corrected. The expression "Hs PARG1" was omitted.

S5: These panels should be shown in the order they are introduced in the main text (e.g., the first ones discussed are panels D – F, which should therefore be A – C). For the current panel A, an extra label "(Met622)" is present on T622M charts, please remove. The figures legend for panel G is labeled as "vii", please fix.
(Answer) The panels were corrected in the order they are introduced in the manuscript. An extra label "(Met622)" was removed and the figure legend for panel G was changed to (**iii**) as the separated section from the C1-RhoGAP complex (**i**) in **Fig. S5**. The map of ϕ-φ angles of Lys^{648} residue was added to section (**iii**) of **Fig. S5**.

1A: Consider adding location of mutations discussed in the work.
(Answer) The position of mutations discussed in this study was added to the schematic representation of PARG1 in **Fig. 1**.

1C, 1D: Should "hydrophilic" in figure legend be "hydrophobic"? Many residues are hydrophobic.
(Answer) The "hydrophilic" was corrected to the "C-terminal" in the figure legend in **Fig. 1c and 1D**.

1E: Please color C1 domain differently than RhoA in Fig. 1D and 1G.

(Answer) The C1 domain was colored as light blue in **Fig. 1D** and **1F** in the **revised Figure 1** (Fig. 1G in previously submitted Figure 1).

1F: X-axis legend should be amino acids of PARG1 (611-886), not construct. Red and brown are hard to differentiate in the figure.

(Answer) Fig. 1F was moved to **Fig. S3b**. X-axis legend was changed to amino acid number of the full-length PARG1 (611-886) and color bars were changed in **Fig. S3b**.

2: Please label some of the regions of the GAP domain, it is difficult to compare to the predicted binding interface shown in **Fig. 1F** (Figure 1G in previous manuscript). Which helices are being shown? Further, what are the black residues; are they necessary to show?

(Answer) The position of highlighted α-helices is indicated in the revised **Figure 2**. Black residues indicate the matched residues between PARG1 and each crystallized GAP structures. This label of crystallized complex was kept and most of the residue information of PARG1 was removed in the revised **Figure 2**.

3C: Please show and label residue I845.

(Answer) The position of I845 residue was labeled in the structure of **Figure 3c.**

4: The figure legend is difficult to parse and some of the information seems like it would be more appropriate to put into the main text. Please also add units to the X-axes (presumably, nanoseconds). There may also be a typo, does the 0.772 Å magnitude refer to a-1 instead of b-3?

(Answer) Units were added to the Z-axes of **Figure 4**. 0.772 Å was referred to **a-2** and average RMSD value of the WT complex **(a-1)** during the stable complex formation (0-30ns) was added to the **Result** section. The part of the figure legend describing the was moved to the **Result section** in the **1st paragraph** of manuscript, and the corresponding sentences in the Result section were divided into two paragraphs.

5A: What does red and blue color coding the upper left panel (initial complex structure) represent? Similar to Fig. 4, some of the information in the legend may be more pertinent to the results section. There is also a typo in legend, Arg625 is listed as “Lys”.

(Answer) The information in the legend and structural images was corrected to be more pertinent to the **Result** sections. The error on the color indication in the upper left panel and Arg^{625} residue was corrected. Note that the **Figure 5a** was revised based on the analysis of 2-μs MD simulation of the WT PARG1 complex.

5B: What does the color coding of the boxes represent? If charge, should histidine be blue?

(Answer) Color coding in **Fig. 5B** was corrected based on RasMol amino colors according to traditional amino acid properties.

6B, 6C: Similar to Fig. 5B, please state what the color codes of the boxes represent (e.g., P36 is orange, but why?). Also, it would be much easier to understand the differences between networks if the residues lined up (e.g., the first residue shown for RhoA in each row is V33, D13, G14, and A15, which is confusing). As it is, one struggles to discern changes in networks should be shown as another panel (i.e., I845V versus pattern B and T622M versus pattern B)?

(Answer) Color codes in **Fig. 6B-6D** in revised version was corrected based on RasMol amino colors. The changes in networks of mutant complexes compared to WT PARG1 complex are also indicated as separated section as **Fig. 6c**.

7A: Please use residue numbers of PARG1 (611-886), not construct.

(Answer) residue number of **Fig. 7A** was changed to that of full-length PARG1.

7B iv: The RMSF scores for T622M look to be higher in C1 domain (orange residues) than shown in 7A iv (RMSF<1). Could the author comment on this discrepancy?

(Answer) The RMSF mapping shown in **Fig. 7B** was the relative RMSF values compared to the average calculated from that of each C1-RhoGAP, so the higher value in the regional domain (orange, red) reflects more dynamic part in the protein. Figure legend in **Fig. 7B** was corrected to avoid the confusion.

8: Please indicate in titles for WT PARG1 which pattern (A or B from Fig. 5) is shown (i.e., for Fig. 8A i and ii), similar to Fig. 7A i and ii.

(Answer) The pattern A or B of WT PARG1 based on the category shown in Fig. 5 is indicated in **Fig. 8A** and **B**.

Tables

S1: Please add residue ranges for PARG1 in legend to match PARG1# and PARG1##.

(Answer) Each residue ranges based on the full-length protein was added to the legend in **Table S1**.
