# Supplementary material for: C1-linker region of PARG1 RhoGAP promotes the catalytic recognition fold of RhoA substrate
Source: PLoS One. 2025 Jul 9;20(7):e0326924. doi: 10.1371/journal.pone.0326924 (PMC12240320; doi:10.1371/journal.pone.0326924)
Supplement: Attachment — Submitted filename: Response to Reviewers.pdf [file pone.0326924.s018.pdf]

Dr. Paul A. Randazzo
Academic Editor
PLoS ONE
Revision of manuscript, submission **ID PONE-D-24-50037R1 [EMID:ea5600c191900474]**

Dear Dr. Randazzo,
Please find attached revised manuscript and marked-up-copy of the manuscript that highlights changes made to the previously submitted manuscript by Kouchi and Kojima entitled "**C1-linker region of PARG1 RhoGAP promotes the catalytic recognition fold of RhoA substrate**", as research article "PLoS ONE". We read carefully your recommendations and the reviewer's criticism and revised the paper accordingly. Here is our point-to-point response to the reviewer's comments, experiment and discussion of all changes in the revised manuscript.

In revised manuscript, all the data obtained by 100-ns MD simulation were removed from **Figure 4-9** and the structural analysis were performed from 2-μs MD simulation by adding the analysis for T622M-RhoA complex. The discussion of AlphaFold application to the GAP complex model and MD analysis for another model (**Figure 1i** in addition to **Figure 1h**) obtained by AlphaFold multimer were also added to the **Discussion part** in the revised manuscript. In the modeled structures using AlphaFold multimer indicated as **Figures 1h** and **1i**, stable complexes were not obtained more than 50-75 ns through long-ranged simulation in the same conditions, presumably due to no interface between the C1 region and RhoA substrate formed. These irreversible dissociation patterns of the complex were not included in the result section, but deduced structural properties of the stable complexes obtained by MD simulation was included as the **Figure S11** and described in the **Discussion** part. The summary of MD analysis for AlphaFold model from the aspects of RhoA interface will be helpful for understanding the dynamics of the GAP fold, and also support the notion that the GAP domain alone is not sufficient for the RhoA interaction. The criteria for selected structure complexes based on 2D-RMSD are included as **Figure 4d-f** and summary of the major conformational changes were included as **Figure 9**.

To Reviewer 1
The authors also explored the conformational changes induced by these mutations and provided insights into how these alterations may affect the GTPase activation process. Despite the novelty and relevance of the study, several methodological and interpretative concerns need to be addressed to strengthen the conclusions drawn and clarify aspects of the experimental approach.
A. Technical approach for the structural data and analysis was clarified in the **Method and Result sections**. **Answers to the concerns for No. 4-6, 9-10, and 13 from reviewer 1 were not included in this form, because all the 100ns-scale MD simulation data were removed from the manuscript and figures, according to the concerns No.6 and 9 by reviewer 1.**

Concerns
1. Clarify whether AlphaFold-Multimer was used for the PARG1-RhoA complex or just single-chain PARG1.
A. PARG1-RhoA complex was modeled using AlphaFold-Multimer and just single-chain PARG1 was

modeled using AlphaFold and subjected to the HDOCK analysis.

The structural model selected for molecular dynamics analysis was based on **template-free mode** of HDOCK analysis, therefore structural data obtained from single-chain PARG1 was examined using the same mode of HDOCK program. In conclusion, the docking analysis of AlphaFold structure model of the single-chain was not consistent with the experimentally validated substrate preference of PARG1 reported by several papers including interactome analysis.

AlphaFold-multimer is one of the available complex analysis tools and selected top model 1-5 were checked, based on the similarity to crystallized RhoGAP-RhoA complexes (PDB: 5c2k, 3msa, 1ow3, 5irc) and interface analysis using RMSD and Drugscore^{PPI}. Model1 and 3 obtained using AlphaFold-multimer (AF-MM) were selected by the similarity to the p190ARhoGAP (p50 and ArhGAP20) and MgcRacGAP, respectively. The description was added to **Result section**.

2. Justify why AlphaFold was chosen and address potential limitations in modeling protein-protein interactions, especially if flexible loops like the C1 domain are involved.

A. Several papers have reported that AlphaFold-mulimer performance was not good without multiple sequence alignment (Strom JM and Luck K (2025) Current Opinion in Struct. Biol. **91**: 103002, Yin et al. (2022) *Prot. Sci.* **31**: e4379). It has been pointed out that domain structures with intrinsic disordered domain (IDR) did not show good scores. We assessed the DockQ scores of the selected five AF-MM PARG1 structural models, and the calculated scores compared to the corresponding selected wild-type PARG1 model containing the C1-loop that was used for 2 μs MD analysis showed 0.345-0.359, with acceptable quality to the modeled structure selected using HDOCK with template-free mode. Detailed description for the reason was added to the **Result section**, pp. 12.

3. Discuss whether AlphaFold's predictions were validated by experimental data (e.g., X-ray, cryo-EM) or alternative structural methods.

A. The predicted errors of regional GAP domain of AlphaFold model were mostly detected in the C-terminal α_9 helix in the x-ray crystallized structures (in the case of 1ow3, 3msx, and 5irc as PDB ID). When compared to the complex models obtained from AlphFold-multimer to the crystallized complexes, the models with minimal RMSD value between 1ow3, 3msx, 5irc, and 5c2k showed higher percentage of cumulative binding free energies of conserved and variable interface residues than those with maximal RMSD value when calculated using Drugscore^{PPI} program. In addition, the proximity of the interface residues of AlphaFold-multimer models were matched to the profiles of crystallized complexes as shown in **Figure S5**. Therefore, we analyzed model 1 and 3 of AlphaFold-multimer, selected based on RMSD values to these experimentally validated structures, for MD simulation. Detailed description was added to the **Result section** (pp. 12, 20) and **Discussion part**.

7. Justify why specific frames were selected for MMPBSA calculations.

A. In 2-μs MD simulation, significant levels of conformational changes were detected and the trajectories were categorized as clusters based on the values of 2D-RMSD. The structural ensembles were classified into 2-8 clusters in each MD simulation based on the differences. Therefore, MMPBSA analysis was performed from the representative structure frames showing the minimal RMSD values within the largest cluster in each RhoA complexes, although the several representative structures were

present in the structural ensembles. This reason was described in **Material and methods and Result parts** (p.15) **with Figure 4**.

8. Clarify the criteria for selecting frames based on 2D-RMSD analysis and explain how these frames represent an equilibrated state of the system.
A. selecting frames based on 2D-RMSD analysis was based on 8 clusters according to the RMSD value as shown in **Figure 4**. In **Figure 9** the representative structures selected from several clusters showing significant conformational changes (i.e. RMSD value: 4-6) were depicted as the superimposed structures. The representative structures (state 2) were selected from the largest clusters during 2-μs MD simulation. Detailed description for producing 2D-RMSD matrices and selecting the representative structures was also added in **Materials and Methods section**.

9. Consider focusing on the 2-μs MD simulation for better insight, as the shorter trajectory may not provide additional significant information.
A. Because 100ns-trajectory data were not sufficient to provide structural variations in this case, we focused on 2-μs MD simulation, and presented the results of MD data in **Figure 4-9 and Table 1** in main text by removing the description on the short trajectory data in this revised manuscript.

12. Integrate the biological significance of the mutations (T622M, I845V) more explicitly. Link the computational findings with experimental data or known functional defects in RhoGAP and RhoA regulation.
A. No biological data has been reported for mutations presented in this manuscript. Therefore, we conducted molecular cloning of the corresponding C1-GAP region (residue number 611-886) of human PARG1 GAP domain from HEK293 cells. Unfortunately, the C1-GAP domain of PARG1 tagged with 3 tandem FLAG epitopes was not exogenously expressed in Neuro2a cells, although the tagged full-length MAGL (monoacylglyecerol lipase, positive control) coding a 33kDa protein was successfully expressed in the same condition (see the following paper as reference: Kouchi Z (2015) *Biophys. Biochem. Res. Communication*, **464**, 603-610). As mentioned before in the previous letter, it was also reported that YFP-tagged PARG1 was poorly expressed in HEK293 cells, which was not sufficient level for calculation of luciferase assay in the following paper (Anderson EL and Hamann MJ, *Biochem. J.* **441**: 869-871, 2012). The comment on the difficulties in cellular studies of PARG1 was included in the **Discussion part** of the revised manuscript. Related references on the variants/mutations were included as previously submitted.

To Reviewer 2
Response to General comment 1: The term “Hydrophilic interface” is still used in a couple cases: figure legend for 3C and 3D; figure legend for 6B. Please make consistent by renaming “C-terminal interface.”
A. The term “Hydrophilic interface” was corrected to the “C-terminal interface” in the figure legends for **Figures 3C and D, and 6B**.

Response to General comment 4: A statement in the Discussion should be made indicating that, while

not examined, it is plausible that the presence of the F-BAR domain and linker region to C1 domain may create steric constraints that cause preference for either pattern A or B as described in the text, or in other words, that the F-BAR domain may disrupt pattern A or B. There is no need to discuss the F-BAR more than that in this context, but it is important to note that its presence may alter the dynamics of the C1 domain relative to the GAP domain and that such a consideration was not in the scope of this work.
A. A statement for the effects of the presence of F-BAR domain was added to the end of **the 6th paragraph** in the Discussion part.

Fig.1E: Please make the C1 domain light blue as seen in panels 1F and 1H. RhoA is depicted in green in all other panels, so the C1 domain should not be green in panel 1E.
A. The C1 domain of **Figure 1E** is labeled in light blue as seen in panels 1F and 1H.

Fig. 5B: One usage of "variable conserved" is shown, please change as this is not intuitive and was largely removed in the most recent version of the manuscript.
A. Usage of "variable conserved" was removed from the revised manuscript in **Figure 5b**.

Fig. 9: Color coding of C1 in WT, I845V, and T622M complex to reduce confusion and the summary in 9B (which should thus become panel D) should be created for I1845V and T622 mutants (as panels E and F).
A. Color coding of C1 in WT, I845V, and T622M were unified with light blue for state 1 and 3, and blue for state2 that are the representative structure selected from the largest cluster in 2D-RMSD. The superimposed structures are separately indicated as **Figure 9a-c**. The summary panel focusing on the map of interface residues in the C1 domain and its residue-residue contact in the Glu^{723} residue located in α_3-helix in each complex was created as **Figure 9d-f** in the revised manuscript.
