# Supplementary material for: C1-linker region of PARG1 RhoGAP promotes the catalytic recognition fold of RhoA substrate
Source: PLoS One. 2025 Jul 9;20(7):e0326924. doi: 10.1371/journal.pone.0326924 (PMC12240320; doi:10.1371/journal.pone.0326924)
Supplement: Attachment — Submitted filename: Review_PLOS.docx [file pone.0326924.s014.docx]

**Reviewer Report**

**Title:**

*Structural Analysis of the PARG1 RhoGAP Domain in Relation to NSCL/P Mutations: A Computational Perspective*

**Summary of the Study**

The manuscript presents an extensive computational investigation into the structural dynamics and functional implications of PARG1 RhoGAP domain mutations (T622M and I845V) implicated in NSCL/P. The author employed advanced computational tools, including homology modeling, molecular docking, and molecular dynamics (MD) simulations, to characterize wild-type (WT) and mutant PARG1 domains. The results suggest that these mutations disrupt the RhoA-binding interface and alter protein stability, providing mechanistic insights into their potential pathogenicity. The use of diverse computational techniques ensures a thorough structural and functional evaluation of WT and mutant PARG1 RhoGAP domains. The manuscript provides an in-depth characterization of key residues, hydrogen bonding networks, and conformational dynamics. However, there are several issues required to be addressed by the author.

**Major comments**:

- The manuscript describes the use of traditional computational techniques, including homology modeling and molecular docking, to model the protein-protein interactions involving the disease-relevant mutations in the PARG1 RhoGAP domain. However, it is notable that the author did not utilize **AlphaFold**, a highly accurate and widely accepted tool for protein structure prediction, particularly for complex structures. The author could have considered using AlphaFold-Multimer to generate the protein-protein complex models, especially for validating the interactions between PARG1 and its partners. This would not only have strengthened their findings but also added confidence to their molecular dynamics simulations, as AlphaFold-generated structures tend to serve as better starting points for downstream simulations due to their higher accuracy.
- The manuscript reports performing MD simulations for 100 ns, which may be considered relatively short for accurately capturing the full scope of protein dynamics and complex interactions, particularly for systems involving protein-protein interactions or allosteric effects. In many cases, **microsecond-scale simulations provide a more comprehensive view of the system's behavior**, allowing for a deeper understanding of long-range conformational changes, transient interactions, and more stable equilibrium states. Protein-protein interactions and allosteric effects, for instance, may not fully manifest over just 100 ns, as significant changes in conformation and the establishment of stable interaction networks can take longer to develop. Rare events, such as the binding/unbinding of ligands or conformational transitions, might require longer simulation times to be adequately sampled.
- While the manuscript provides a solid computational analysis of the structural changes caused by the T622M and I845V mutations, it lacks a detailed discussion on how these findings correlate with published biological data. The connection between these computational observations and known experimental studies on the effects of similar mutations in RhoGAP proteins (or homologous proteins) is not adequately addressed. Mutations in the RhoGAP domain are known to affect the regulation of RhoA GTPases, a key player in cellular processes such as migration and cytoskeletal organization. Including references to experimental studies linking RhoGAP mutations to cellular dysfunction would strengthen the manuscript and provide biological context.
- The manuscript introduces an intriguing link between PARG1 RhoGAP mutations and NSCL/P, but the connection remains speculative. The discussion could benefit from more focus on the developmental biology of cleft lip and palate and how RhoA signaling is involved in craniofacial development. Altered cellular dynamics resulting from disrupted RhoA regulation are likely contributors to developmental disorders, but this connection is not sufficiently explored.
- MMPBSA analysis is a useful tool for estimating the binding free energy differences between mutant and wild-type protein-protein complexes. In the context of the T622M and I845V mutations, this method could help quantify how these mutations affect the interaction stability between PARG1 and RhoGAP, potentially linking changes in interaction energy to disease-causing effects.
- The RMSD plots presented in the manuscript lack clarity regarding their generation, raising questions about the apparent discontinuities. These breaks could result from improper post-processing of trajectory data, potentially due to periodic boundary conditions (PBC) not being correctly accounted for during the RMSD calculations. Proper re-centering and imaging should be performed to ensure a continuous and accurate trajectory representation. Furthermore, the figure legends are overly descriptive but fail to provide sufficient explanation for critical features in the plots. For instance, the annotations "1," "2," and "3" in the RMSD plots are introduced without a clear description of what they signify. The author should revise the legends to concisely explain these elements and ensure the figures are intuitive and informative.

**Minor Comments**

- Several sections in the manuscript contain repetitive information, especially regarding the interactions of specific residues with RhoA and their role in protein stability. This redundancy can detract from the clarity of the manuscript.
- The figures are well-designed and aid in understanding the computational results. However, some figure legends are repetitive and could be streamlined to highlight the most important findings.
- Y-axis label units (Angstroms??) are missing in Fig. 8a.
