# Supplementary material for: C1-linker region of PARG1 RhoGAP promotes the catalytic recognition fold of RhoA substrate
Source: PLoS One. 2025 Jul 9;20(7):e0326924. doi: 10.1371/journal.pone.0326924 (PMC12240320; doi:10.1371/journal.pone.0326924)
Supplement: Attachment — Submitted filename: Review.docx [file pone.0326924.s016.docx]

The manuscript presents a computational analysis of the structural dynamics of **PARG1**, a **RhoGAP protein**, and its interactions with **RhoA**, focusing on the effects of the **T622M** and **I845V** mutations. The authors used a combination of structural modeling, **molecular dynamics (MD) simulations**, and **binding free energy calculations** (via **MMPBSA**) to investigate the recognition mechanisms of PARG1 and how these mutations disrupt its interaction with RhoA. The authors also explored the conformational changes induced by these mutations and provided insights into how these alterations may affect the **GTPase activation** process. Despite the novelty and relevance of the study, several methodological and interpretative concerns need to be addressed to strengthen the conclusions drawn and clarify aspects of the experimental approach.

Concerns:

- Clarify whether AlphaFold-Multimer was used for the PARG1-RhoA complex or just single-chain PARG1.
- Justify why AlphaFold was chosen and address potential limitations in modeling protein-protein interactions, especially if flexible loops like the C1 domain are involved.
- Discuss whether AlphaFold’s predictions were validated by experimental data (e.g., X-ray, cryo-EM) or alternative structural methods.
- Discontinuities in the RMSD graphs should be addressed—likely due to improper handling of periodic boundary conditions (PBC).
- The 2-µs simulation shows RMSD up to 10 Å, which is drastically higher than the 100-ns simulation (3 Å). Explain why both short and long simulations are compared and whether they are directly comparable.
- Ensure RMSD graphs are properly processed, including re-centering and imaging of the trajectories to handle PBC. Consider focusing on only one longer simulation (e.g., 2-µs MD) for better accuracy.
- Justify why specific frames (e.g., 15-30ns for WT PARG1, 15-45ns for p.Ile845Val) were selected for MMPBSA calculations.
- Clarify the criteria for selecting frames based on 2D-RMSD analysis and explain how these frames represent an equilibrated state of the system.
- The terms Pattern A and Pattern B are introduced but not sufficiently explained. Clarify what these patterns represent (e.g., specific conformational states, structural transitions, or interaction patterns).
- Provide a clear definition of Pattern A and Pattern B, along with how they relate to the simulation dynamics and protein-protein interactions.
- Consider focusing on the 2-µs MD simulation for better insight, as the shorter trajectory may not provide additional significant information.
- Integrate the biological significance of the mutations (T622M, I845V) more explicitly. Link the computational findings with experimental data or known functional defects in RhoGAP and RhoA regulation.
- Simplify and clarify figure legends, particularly explaining the meaning of labels like “1”, “2”, and “3” in the RMSD plots.
