# Supplementary material for: C1-linker region of PARG1 RhoGAP promotes the catalytic recognition fold of RhoA substrate
Source: PLoS One. 2025 Jul 9;20(7):e0326924. doi: 10.1371/journal.pone.0326924 (PMC12240320; doi:10.1371/journal.pone.0326924)
Supplement: Attachment — Submitted filename: Review-letter- to-Editor.docx [file pone.0326924.s017.docx]

**Review of Manuscript: C1-linker region of PARG1 RhoGAP promotes the catalytic recognition fold of RhoA substrate**

Dear Editor,

I have reviewed the revised version of the manuscript entitled "C1-linker region of PARG1 RhoGAP promotes the catalytic recognition fold of RhoA substrate" by Zen Kouchi and Masaki Kojima, which investigates the structural dynamics and protein-protein interactions of PARG1, a RhoGAP protein, with RhoA. While the authors have addressed some of the earlier concerns, several significant issues remain unaddressed or inadequately addressed, leading me to recommend rejection of the manuscript in its current form. Below are the major concerns:

1. While the authors have now utilized AlphaFold for modeling the PARG1 structure, it is unclear whether the AlphaFold-Multimer was used to model the PARG1-RhoA complex or just individual proteins. Given the central role of protein-protein interactions in this study, using AlphaFold-Multimer would have been more appropriate for predicting complex structures, especially considering the flexible loops like the C1 domain. However, the authors do not provide sufficient justification for their choice of modeling method, and the limitations of using AlphaFold in modeling such interactions are not adequately discussed. The authors need to clarify whether the AlphaFold predictions have been validated through experimental data or any other structural techniques (e.g., X-ray crystallography or cryo-EM) to ensure the accuracy of their models.

2. The RMSD graphs presented in the manuscript show significant discontinuities, which likely arise from improper handling of periodic boundary conditions (PBC) during the simulation. The authors do not address this issue or explain how the RMSD values were processed, which raises concerns about the reliability of the results. Additionally, the 2-µs MD simulation exhibits RMSD values up to 10 Å, which is far higher than the 100-ns simulation (~3 Å). The authors should explain why they compared simulations of two different lengths, as these results cannot be directly compared due to the significant time disparity. Furthermore, the drastic discrepancy in RMSD between the simulations warrants further clarification, especially regarding whether the 100-ns data adequately captured the relevant dynamics of the system.

3. The authors mention using 2D-RMSD analysis to select representative frames for MMPBSA calculations, but they fail to provide a clear justification for their selection process. For instance, why were frames like 15-30ns for wild-type (WT) PARG1, and 15-45ns for the I845V and T622M mutants chosen? The authors need to clarify how these specific frames were selected based on their 2D-RMSD analysis, and whether they represent equilibrated states of the system. Without a clear rationale for frame selection, the reliability of the binding free energy calculations remains questionable.

4. The terms Pattern A and Pattern B are introduced but are not clearly defined or explained. The manuscript mentions that these patterns represent different C1-loop orientations, but the explanation lacks sufficient clarity. The authors should provide a more detailed description of what Pattern A and Pattern B specifically represent in terms of structural states and dynamics of the C1 domain. This lack of clarity complicates the understanding of the figures and the conclusions drawn from the analysis.

5. The manuscript uses both 100-ns MD and 2-µs MD simulations to analyze the structural dynamics of PARG1 and its mutants. However, if the 2-µs simulation offers a more comprehensive analysis and the shorter simulation may not provide significant additional insights compared to the longer simulation. The authors should justify the use of both simulation lengths, or ideally, focus on the 2-µs MD simulation to provide a more accurate and robust dataset.

6. While the manuscript addresses the structural impact of the T622M and I845V mutations, the biological relevance of these mutations remains underexplored. The authors should more explicitly connect their computational findings to experimental studies and known biological consequences of these mutations, particularly in the context of RhoGAP function and RhoA regulation. For example, linking these mutations to known cellular dysfunctions, such as altered migration or cytoskeletal reorganization, would strengthen the manuscript's biological relevance.

7. The figure legends in the manuscript are overly descriptive but lack clarity in explaining the critical aspects of the data, particularly in relation to the RMSD plots. For example, the labels “1”, “2”, and “3” in the RMSD plots are not explained in sufficient detail, leaving readers unclear about their significance. The authors should revise the figure legends to clearly explain the data, including the meaning of these labels, and ensure that the figures are presented in a more intuitive and informative manner.

Altogether, while the study presents a valuable attempt to model and analyze the structural dynamics of PARG1-RhoA interactions, the manuscript contains several significant issues that undermine its conclusions. These issues range from inadequate clarification of modeling techniques and simulation analysis to unclear data representations and a lack of sufficient biological context. Therefore, I recommend rejection of the manuscript in its current form. The authors should address these major concerns thoroughly before resubmitting the manuscript for further consideration.

Sincerely,
Venkata Chirasani
